# Modulator-induced conformational changes in complement C5, implications for function and drug design

**DOI:** 10.3389/fimmu.2026.1834455

**Published:** 2026-05-15

**Authors:** Andrey Zhivnov, Carolina Ottochian, Karim El-Bouri, Rose Francis, Rosie Campbell, Alex Macpherson, Stephen Wells, Jean MH van den Elsen

**Affiliations:** 1 Department of Life Sciences, University of Bath, Bath, United Kingdom; 2 Protein Biogenesis Laboratory, Francis Crick Institute, London, United Kingdom; 3 Immunology Therapeutic Area, UCB Pharma, Slough, United Kingdom; 4 Department of Chemistry, University of Bath, Bath, United Kingdom

**Keywords:** allosteric inhibition mechanism, C5 convertase, complement C5, complement inhibition, innate immunity, protein conformational analysis

## Abstract

**Background:**

The human complement system comprises proteolytic cascades that drive protective inflammatory and cytolytic responses, making it a crucial part of innate immunity. Complement component C5 links the upstream activation pathways to the terminal pathway, and its cleavage by classical and alternative pathway C5 convertases generates potent effectors implicated in autoimmune and inflammatory diseases. Several inhibitors targeting diverse sites on C5 are in clinical use or development. However, the structural basis by which binding at distinct sites achieves comparable functional outcomes remains poorly understood.

**Methods and results:**

We systematically compare twelve C5-modulator complexes using domain superposition, interface quantification, C5a scissile loop conformational analysis, and rigid cluster decomposition. We demonstrate that modulators propagate conformational and dynamic changes well beyond their binding sites, identifying the MG3 domain as a conformational relay that transmits perturbations across the C5 scaffold and MG8 as a domain whose rigidity correlates with global constraint redistribution. Structurally unrelated inhibitors converge on α-helical restructuring of the normally disordered C5a scissile loop, although this feature is neither necessary nor sufficient for complete pathway blockade. Benchmarking against the cobra venom factor (CVF)-bound structure as a proxy for C5 convertase engagement, we show that complete inhibitors, notably eculizumab and the tick-derived OmCI-RaCI complexes, achieve efficacy through distinct structural rearrangements rather than a single shared mechanism, whereas partial inhibitors each lack a different subset of these properties.

**Conclusions:**

These findings establish that C5 inhibition is governed by the nature and extent of conformational perturbation rather than by simple steric hindrance of convertases alone, providing a structural framework for rational design of next-generation complement therapeutics.

## Introduction

**Figure 1:**
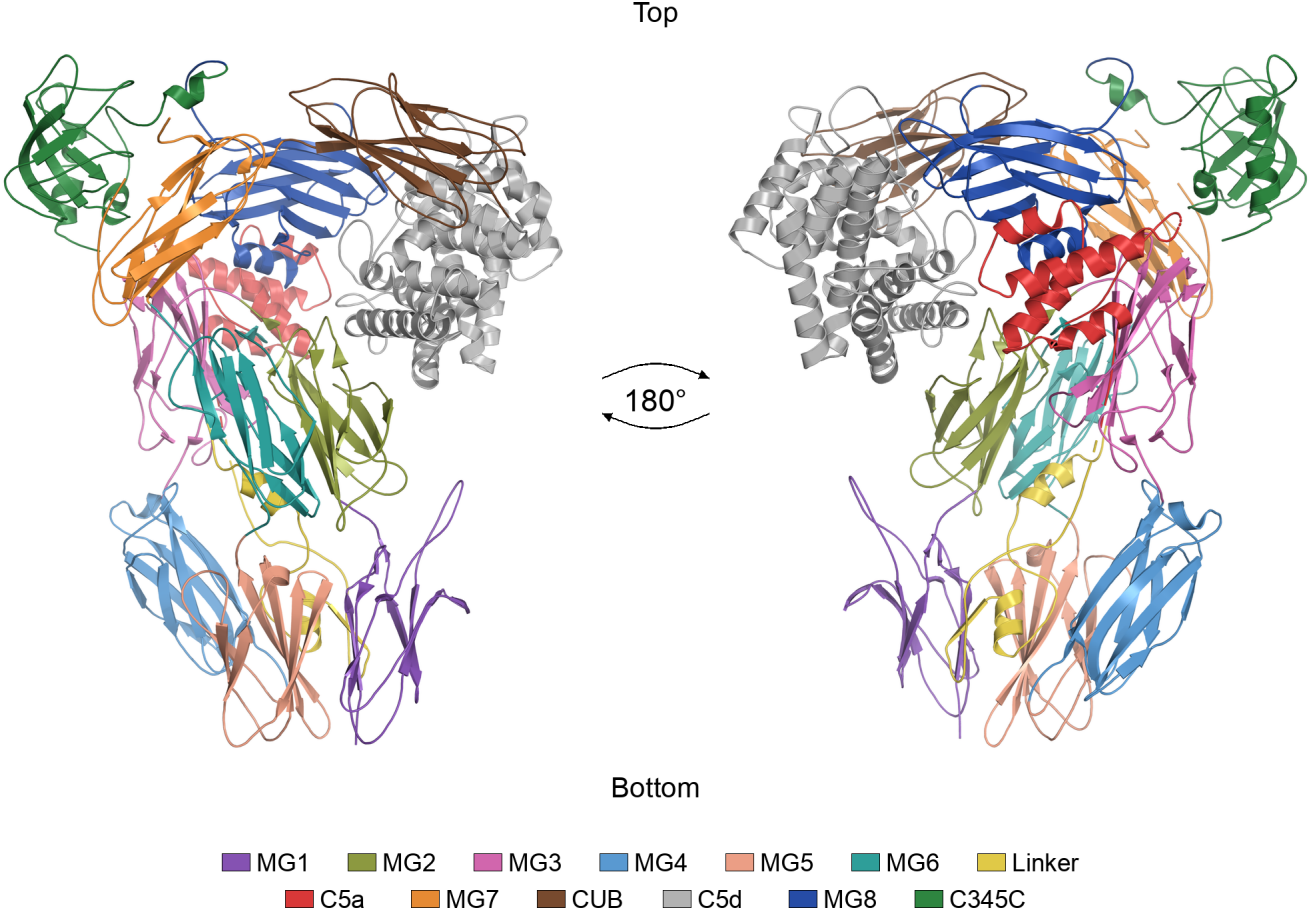
Domain architecture of complement component C5. Cartoon representation of apo C5 (PDB: 3CU7, chain A), shown in two orientations related by a 180° rotation about the vertical axis, with the “top” and “bottom” faces indicated. The domains are coloured as follows: MG1 (residues 20-124), MG2 (125-223), MG3 (224-351), MG4 (352-459), MG5 (460-565), MG6 (566-613 and 760-822), linker (614-673), C5a (679-759), MG7 (823-931), CUB (932-982 and 1308-1371), C5d (983-1307), MG8 (1372-1521), and C345C (1522-1676). Domain boundaries were assigned from CATH annotations (14) as described in Materials and methods.

The human complement system is an essential part of innate immunity, comprising a hierarchical proteolytic cascade that facilitates pathogen opsonisation, chemotactic signalling and direct cell lysis (1). Its activation can proceed via the classical (CP), lectin (LP), or alternative (AP) pathways, all of which converge on the cleavage of complement component 3 and 5 (C3 and C5) by bi- and trimolecular convertases (C3: C4b2a in CP and LP, C3bBb in AP; C5: C4b2a3b in CP and LP, C3bBb3b in AP, respectively). Although recent structural advances have characterised C3 convertase activity and C3-substrate recognition in CP (2), mechanistic understanding of C5 convertase function remains limited. Structurally, C5 is a large (~190 kDa) glycosylated heterodimer composed of an N-terminal β-chain and a C-terminal α-chain, covalently tethered by a disulfide bond (3). The β-chain comprises domains MG1-MG5, the N-terminal segment of MG6, and the linker region, while the α-chain comprises the C-terminal segment of MG6 along with C5a, MG7, CUB, C5d, MG8, and C345C domains. Spatially, domains MG1-MG6 form a right-handed “superhelix” connected via the MG7 domain to a “superdomain” comprising CUB, C5d, and MG8 domains. Within the superhelix, MG1 forms an interface with MG5 and the linker, forming the “bottom face” of C5. The MG4 domain forms the structural centre of the superhelix, interacting with both MG3 and MG5 domains while providing a cavity to accommodate the linker. Besides interacting with MG3, the MG2 domain directly contacts C5d, a domain structurally homologous to the thioester-containing domain of C3 and C4, but lacking the reactive internal thioester bond. The MG7 domain acts as a hinge to the superdomain, where the split CUB domain interacts with MG8 and C5d, which together with C345C form the “top face” of C5 (**Figure 1**).

The C5a domain adopts a four-helix bundle fold, containing a loop (known as the “scissile loop”) that harbours the cleavage site (Arg751-Leu752 peptide bond, also known as the “scissile bond”) for C5 convertases. The linker, characterised by two α-helical elements, threads through the centre of the superhelix, restraining the C5a domain within the split MG6 domain. This constrained topology sterically shields the scissile bond, preventing spontaneous activation and ensuring C5 remains kinetically inert until engaged by the convertase (4). Mechanistically, it has been proposed that the auxiliary C3b subunit within the C5 convertases functions as a high affinity allosteric exosite that captures native C5 to overcome the intrinsic steric resistance of the substrate to proteolysis. This interaction orients the scissile bond toward the catalytic serine protease subunit C2a or Bb within the C5 convertases, allowing its hydrolysis to generate C5a and C5b fragments (5). C5a acts as a potent anaphylatoxin primarily via the C5aR1 receptor (6), while C5b undergoes major conformational changes to facilitate the nucleation of the membrane attack complex (MAC), initiating the terminal complement pathway (7).

Given the central role of C5 in the terminal complement pathway, many pathogens have evolved mechanisms to inhibit its cleavage, aiming to suppress complement-mediated immune response. For example, hematophagous ticks, such as *Ornithodoros moubata*, *Rhipicephalus appendiculatus*, and *Rhipicephalus pulchellus* evolved distinct salivary proteins which function as potent C5 inhibitors. These are OmCI, RaCI (8), and CirpT (9), respectively, which bind C5 at distinct sites. On the other hand, bacterial immune evasion is exemplified by the *Staphylococcus aureus* superantigen-like protein 7 (SSL7), which binds to and inhibits C5 via its C-terminal β-grasp domain and cooperative recruitment of IgA (10). Interestingly, bovine antibody-derived knob domain peptides K8 and K92 were also shown to bind and inhibit C5 (11). From a therapeutic perspective, dysregulated complement activation underlies several serious conditions, including paroxysmal nocturnal haemoglobinuria (PNH), atypical haemolytic uremic syndrome (aHUS), and myasthenia gravis (MG). These diseases, which can be life-threatening without treatment, have made C5 a prime therapeutic target. This led to the development of an FDA-approved anti-C5 monoclonal antibody eculizumab (4) and the experimental small molecule inhibitor H1H (12).

By contrast, cobra venom factor (CVF) functions as a C5 activator (5). Structurally, CVF resembles C3b, allowing it to directly bind C5 and potentially form a convertase complex with the catalytic subunits C2a or Bb. Therefore, the C5-CVF complex is thought to resemble the C5-convertase interaction the closest. Furthermore, inhibitors sharing the interaction surfaces on C5 with CVF are interpreted to inhibit C5 by sterically hindering the convertase, whereas the ones binding at sites different from CVF are thought to act allosterically (8). Specifically, CVF binds the MG4/MG5 interface and the MG7 domain of C5. Consequently, CirpT variant 1 (CirpT1), which binds MG4, and eculizumab, which binds MG7, likely inhibit C5 by sterically hindering the convertase. Meanwhile, OmCI, RaCI variant 1 (RaCI1), RaCI variant 2 (RaCI2), RaCI variant 3 (RaCI3), K8, K92, and H1H bind other regions and thereby likely inhibit C5 via an allosteric mechanism. An exception is perhaps SSL7, which primarily binds the MG1/MG5 interface, however its subsequent recruitment of IgA suggests that it inhibits C5 by steric hindrance of convertase binding. Despite that, the isolated SSL7 β-grasp domain can bind and inhibit C5, suggesting the potential existence of an allosteric mechanism (10). Moreover, C5 inhibitors can be differentiated in their ability and extent of CP/AP inhibition. All C5 structures analysed in this study, including their structural and functional characteristics, are summarised in **Table 1**.

**Table 1:** Structural and functional characteristics of C5 structures.

C5 structure	PDB	Resolution (Å)	Modulator	Binding site	C345C domain	AP inhibition	CP inhibition
Apo C5 (3)	3CU7	3.10	-	-	Yes	-	-
C5-CVF (5)	3PVM	4.30	CVF	MG4/MG5/MG7	Yes	-	-
C5-CVF-SSL7 (5)	3PRX	4.30	CVF; SSL7	MG4/MG5/MG7; MG1/MG5	Yes	-; Yes	-; Partial
C5-CVF-H1H (12)	8AYH	3.35	CVF; H1H	MG4/MG5/MG7; C5a/MG8	No	-; Yes	-; Yes
C5-OmCI-RaCI1 (8)	5HCE	3.12	OmCI; RaCI1	C5d/CUB; C5d/MG1/MG2	Yes	Yes	Yes; Yes
C5-OmCI-RaCI2 (8)	5HCD	2.98	OmCI; RaCI2	C5d/CUB; C5d/MG1/MG2	Yes	Yes	Yes; Yes
C5-OmCI-RaCI3 (8)	5HCC	2.59	OmCI; RaCI3	C5d/CUB; C5d/MG1/MG2	Yes	Yes	Yes; Yes
C5-OmCI-RaCI1-CirpT1 (9)	6RQJ	3.50	OmCI; RaCI1; CirpT1	C5d/CUB; C5d/MG1/MG2; MG4	Yes	Yes; Yes; Yes	Yes; Yes; Yes
C5-Eculizumab (4)	5I5K	4.20	Eculizumab	MG7	Yes	Yes	Yes
C5-K8 (11)	7AD7	2.30	K8	MG8	No	Partial	Partial
C5-K92 (11)	7AD6	2.75	K92	MG1/MG5	Yes	Partial	No
C5-SSL7 (10)	3KLS	3.60	SSL7	MG1/MG5	Yes	Yes	Partial
C5-SSL7 β-grasp domain (10)	3KM9	4.20	SSL7 β-grasp domain	MG1/MG5	No	Yes	Partial

Summary of C5 structures analysed in this study, comprising the apo C5 reference structure and twelve C5-modulator complexes. For each structure, the PDB identifier, crystallographic resolution, bound modulator(s), and corresponding binding site(s) on C5 are listed. For complexes containing multiple modulators, entries are separated by semicolons in corresponding order. The C345C domain column indicates whether the C345C domain was resolved in the crystal structure (“Yes”) or could not be resolved (“No”). The alternative pathway (AP) and classical pathway (CP) inhibition columns indicate the inhibitory activity of each modulator against the respective complement pathway, where “Yes” denotes complete inhibition, “Partial” denotes partial inhibition, and “No” denotes no inhibitory effect. CVF (cobra venom factor) functions as a C5 activator rather than an inhibitor and is therefore listed as “-” for both pathways.

Biological macromolecular complexes have been extensively studied using a range of high-resolution structural techniques, such as X-ray crystallography and cryo-electron microscopy (13). For experimentally resolved protein structures, the Protein Data Bank (PDB) serves as a publicly accessible repository that archives coordinate files, which constitute the primary structural data used for subsequent analysis. Meanwhile, the advancing field of computational biology enables the rapid and precise manipulation of biological structures. Leveraging these computational approaches, we aim to conduct an in silico structural analysis of C5 inhibition. We achieve this by conducting a series of analyses on twelve C5-modulator complexes while using the structure of apo C5 as a reference (**Table 1**). We first quantify domain displacement by superimposing individual C5 domains of C5-modulator complexes onto apo C5 to determine changes in distance and rotation. We then measure the domain interface surface area of domains that show the most notable differences in domain-by-domain alignment, further characterising how modulators alter their interaction. Next, we assess the effect of C5 modulators on the conformation of the scissile loop and further investigate the interaction changes within the C5a domain. Lastly, we examine the dynamics of C5 structures by applying rigid cluster decomposition (RCD) to determine how modulators redistribute structural constraints across C5.

## Materials and methods

### Structure selection and preparation

Structures of apo C5 and C5-modulator complexes analysed in this study are listed in **Table 1**. Coordinate files were obtained from the Protein Data Bank and visualised in PyMOL (Schrödinger, LLC). For structures containing multiple copies of the C5 complex in the asymmetric unit, one copy was retained and all others deleted. Additionally, all modulator structures, ligands, ions, and solvent molecules were removed, such that each processed file contained a single C5 structure. Domain boundaries were defined using CATH annotations (14) for apo C5 and applied consistently across all structures: MG1 (residues 20-124), MG2 (125-223), MG3 (224-351), MG4 (352-459), MG5 (460-565), MG6 (566-613 and 760-822), linker (614-673), C5a (679-759), MG7 (823-931), CUB (932-982 and 1308-1371), C5d (983-1307), MG8 (1372-1521), and C345C (1522-1676). The C345C domain was not resolved in C5-CVF-H1H, C5-K8, and C5-SSL7 β-grasp domain structures and was therefore excluded from analyses involving these complexes.

### Domain displacement analysis

Domain displacement in C5-modulator complexes relative to apo C5 was quantified using a standalone Python script (available at Zenodo upon publication) implementing a domain-by-domain superposition approach. Cα atom coordinates were extracted from PDB coordinate files for apo C5 and each C5-modulator complex. For every complex, each domain was individually superimposed onto the corresponding domain of apo C5 using the Kabsch algorithm (15), which computes the optimal rotation matrix minimising the root-mean-square deviation (RMSD) between matched Cα atoms. Following each superposition, the displacement of all remaining non-superimposed domains relative to their positions in apo C5 was measured. Translational displacement was defined as the Euclidean distance (Å) between the centre of mass (COM) of a given domain in the transformed C5-modulator complex and the COM of the same domain in apo C5, where COM was calculated as the arithmetic mean of domain Cα coordinates. Rotational displacement was defined as the angle (°) between two vectors originating from the COM of the superimposed domain: one extending to the COM of a given domain in the transformed complex, and one extending to the COM of the same domain in apo C5. This procedure was repeated iteratively using each apo C5 domain as the superposition reference.

### Domain interface analysis

Domain interface surface areas (Å²) were quantified using PDBePISA (16). For every structure, each domain was assigned a unique chain identifier using the “alter” command in PyMOL. The domain interface surface area for MG3 (with MG2, MG4, MG6, C5a, MG7, and MG8), MG4 (with MG3, MG5, and linker), and C5a (with MG3, MG6, C5d, and MG8) was calculated as the sum of the buried surface areas between two given domains. The changes in domain interface surface areas were calculated relative to apo C5.

### C5a domain analysis

C5a domain scissile loop conformation was determined using MolProbity (17). For every structure, backbone dihedral angles (φ and ψ) were extracted for each residue comprising the C5a domain scissile loop (Asp746-Met754), and a Ramachandran plot analysis was conducted. Scissile loop structures were additionally visualised in PyMOL for verification. To characterise intramolecular interactions involving scissile loop residues, hydrogen bonds and salt bridges at the C5a/MG3 interface were identified in PyMOL using polar contact analysis with default distance cut-offs.

### Rigid cluster decomposition analysis

Rigid cluster decomposition (RCD) was performed using the FLEXOME software suite (18) to characterise the distribution of structural constraints across all C5 structures. FLEXOME implements the pebble-game rigidity analysis (19) with the same algorithm as the FIRST (Floppy Inclusions and Rigid Substructure Topography) software (20) and an improved identification of strong salt bridge interactions (21). Specifically, FLEXOME constructs a constraint network from covalent and non-covalent bonding interactions identified by interatomic geometry. Each constraint type is assigned an integer value (“bars”) reflecting its mechanical contribution: six bars for locked backbone dihedrals, five bars for polar hydrogen bonds and rotatable dihedrals, and two bars for hydrophobic tethers. The pebble-game algorithm (19) assigns degrees of freedom to each atom and tests each constraint by searching for available degrees of freedom, identifying constraints as either independent (where degrees of freedom can be mobilised) or redundant (where they cannot). Regions connected by redundant constraints are grouped into rigid clusters (sets of atoms that move as a single rigid body) while regions retaining independent constraints remain flexible. Prior to analysis, hydrogen atoms were added to all structures using REDUCE (22), with optimisation of Asn, Gln, and His sidechain orientations to maximise hydrogen bonding potential. For every structure, RCD was performed at hydrogen bond energy cut-offs of -0.5, -1.0, -1.5, and -2.0 kcal/mol to assess the relative strength of constraint networks; regions retaining rigidity at the more negative cut-offs represent stronger networks, while those rigid only at less negative cut-offs depend on weaker hydrogen bonds. Cluster sizes were ranked by total atom count, and rigidity was quantified as the percentage of domain Cα atoms assigned to one of the twenty largest rigid clusters. Structures were visualised in PyMOL as cartoon representations, with domains coloured by percentage rigidity retention: grey (0%), light blue (1-49%), and purple (≥50%), with colour intensity increasing proportionally within each range.

## Results

### C5-modulator complexes show substantial domain rearrangements

**Figure 2:**
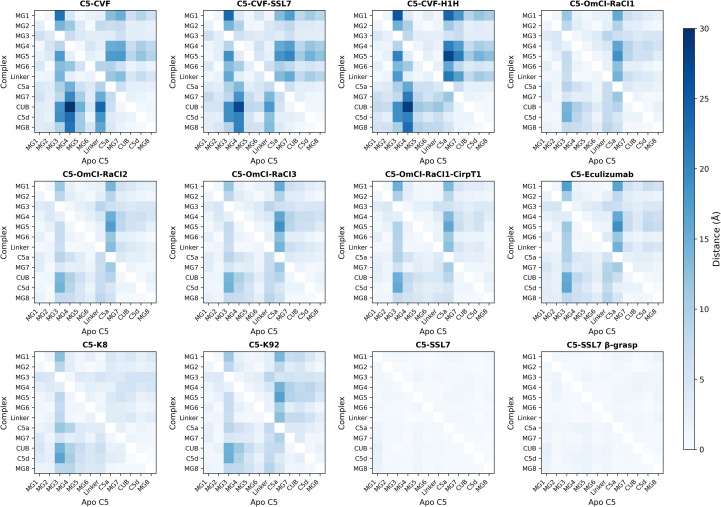
Translational displacement of C5 domains in C5-modulator complexes relative to apo C5. Heatmaps showing centre-of-mass translational displacement (Å) of individual C5 domains in each C5-modulator complex, obtained by domain-by-domain alignment to apo C5. Rows represent complex domains superimposed onto apo C5 domains (columns). Colour saturation increases with displacement magnitude (0-30 Å). The C345C domain was excluded as it could not be resolved in C5-CVF-H1H, C5-K8, and C5-SSL7 β-grasp domain, and shows substantially greater displacement in most other complexes.

**Figure 3:**
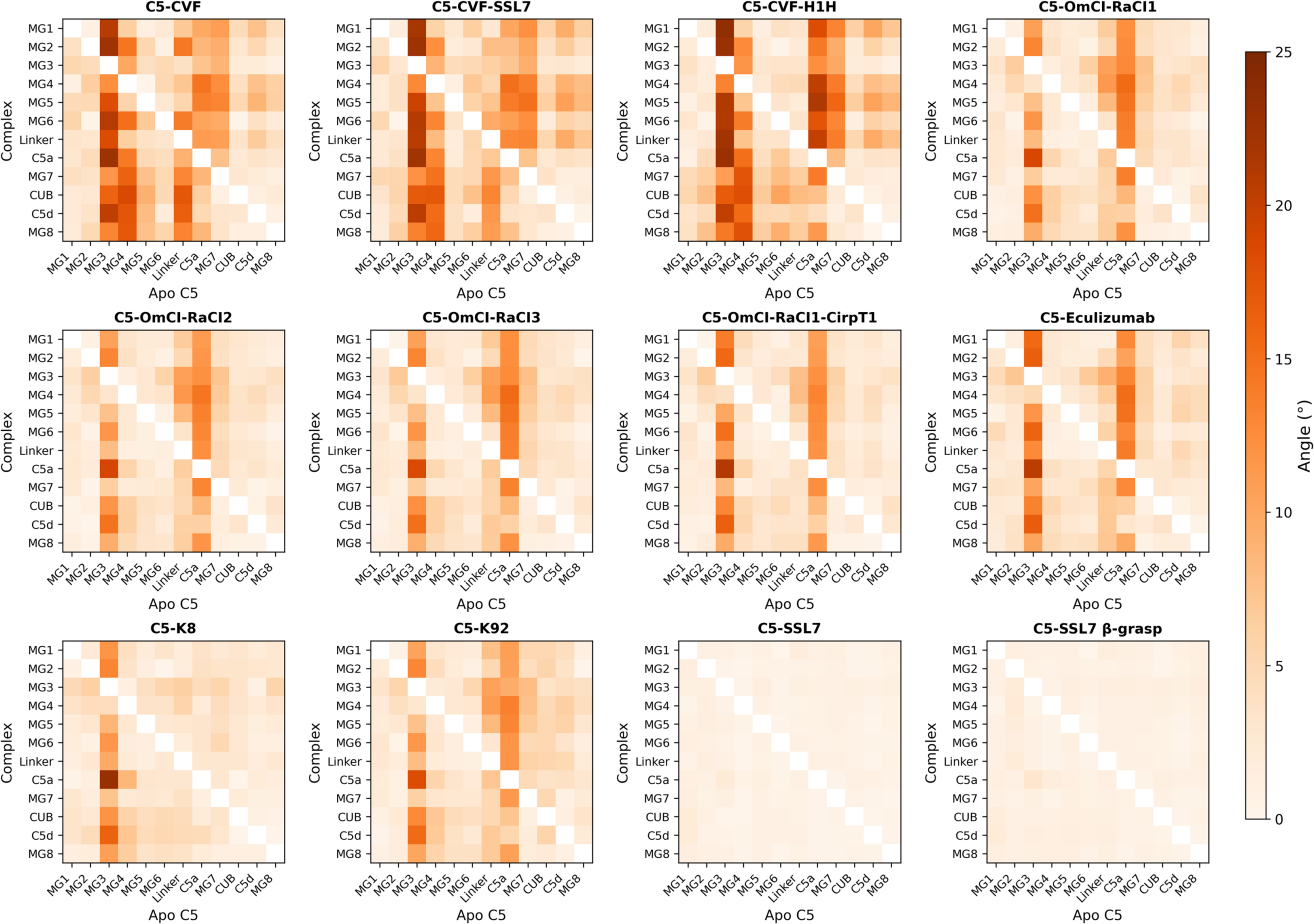
Rotational displacement of C5 domains in C5-modulator complexes relative to apo C5. Heatmaps showing centre-of-mass rotational displacement (°) of individual C5 domains in each C5-modulator complex, obtained by domain-by-domain alignment to apo C5. Rows represent complex domains superimposed onto apo C5 domains (columns). Colour saturation increases with displacement magnitude (0-25°). The C345C domain was excluded as it could not be resolved in C5-CVF-H1H, C5-K8, and C5-SSL7 β-grasp domain, and shows substantially greater displacement in most other complexes.

To evaluate how C5 modulators affect the conformation of C5, we calculated the translational (Å) and rotational (°) displacement of each C5 domain in each C5-modulator complex relative to apo C5 (**Figures 2**, **3**; **Supplementary Tables 1**-**12**). This analysis revealed that most C5-modulator complexes display substantial domain rearrangements. The C5-SSL7 and C5-SSL7 β-grasp domain complexes were the only exceptions, showing negligible translational and rotational displacement.

**Figure 4:**
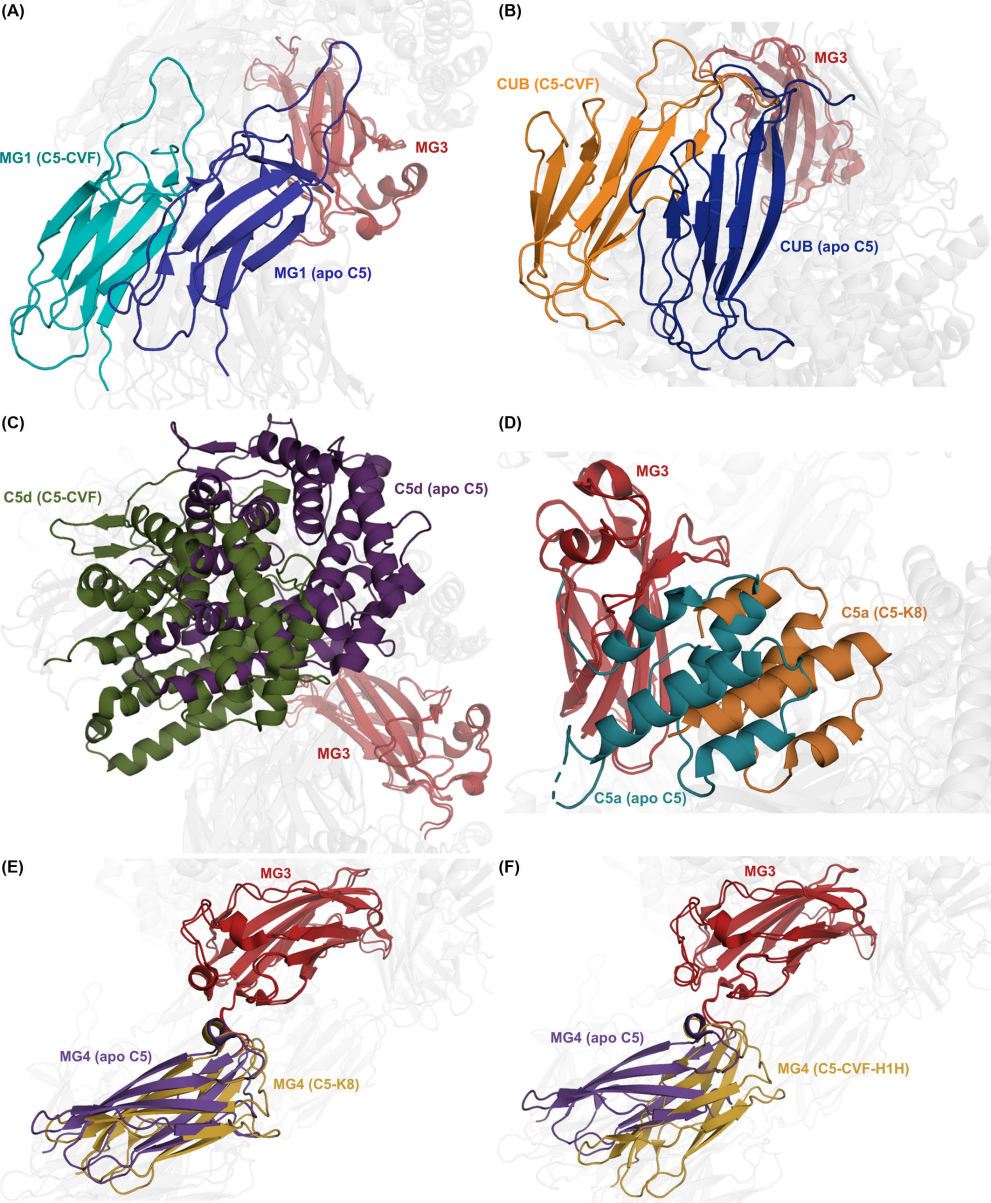
Domain displacement in selected C5-modulator complexes upon superposition on MG3 relative to apo C5. **(A)** Positional shift in C5-CVF MG1 domain (apo C5, deep blue; C5-CVF, teal) upon superposition on MG3 (dark red), showing a 22.6 Å and 20.8° displacement. **(B)** Positional shift in C5-CVF CUB domain (apo C5, dark blue; C5-CVF, orange) upon superposition on MG3 (dark red), showing an 18.3 Å and 16.1° displacement. **(C)** Positional shift in C5-CVF C5d domain (apo C5, plum; C5-CVF, olive) upon superposition on MG3 (dark red), showing a 19.0 Å and 20.3° displacement. **(D)** Positional shift in C5-K8 C5a domain (apo C5, turquoise; C5-K8, orange) upon superposition on MG3 (dark red), showing an 11.6 Å and 22.9° displacement. **(E, F)** Positional shift in **(E)** C5-K8 and **(F)** C5-CVF-H1H MG4 domain (apo C5, purple; complex, gold) upon superposition on MG3 (dark red), showing a 3.0 Å and 4.3° displacement and a 9.1 Å and 13.3° displacement, respectively.

In particular, we observed that the MG3 domain undergoes a significant positional shift in most complexes relative to apo C5, evidenced by large translational and rotational deviations in multiple domains when structures are superimposed on MG3. For example, in C5-CVF, MG1 undergoes a 22.6 Å distance shift, accompanied by a 20.8° rotation relative to its position in apo C5 (**Figure 4A**). Similarly large rearrangements are observed for the CUB and C5d domains (**Figures 4B, C**). The C5-CVF-SSL7 and C5-CVF-H1H complexes show comparable conformational change patterns, with MG1 relative displacements of 25.6 Å and 23.5° in C5-CVF-H1H. The C5a domain is likewise repositioned in most C5-modulator complexes, exhibiting a notable rotation upon superposition of MG3 (up to 22.9° in C5-K8; **Figure 4D**). Notably, MG4 virtually retains its position relative to MG3, with the most significant displacement in CVF-bound structures, where MG4 is translated by 8-9 Å and rotated by 12-13° (**Figures 4E, F**).

By contrast, superimposing MG7 shows that CUB, C5d, and MG8 remain closely aligned across all complexes, with displacement <3 Å and <4°, relative to apo C5. Likewise, superimposing C5d shows minimal relative displacement of CUB and MG8, with translations ≤1 Å and rotations ≤1.5° in most complexes. Moreover, superimposing MG4 shows that MG1 and MG5 remain closely aligned in all complexes, evidenced by relative displacement <4 Å and <5° in non-CVF complexes, with slightly larger displacement (up to 4.5 Å and 7.4°) in CVF-bound structures. Notably, in complexes where C345C is resolved and except in C5-SSL7, this domain shows the largest relative displacement when aligned to any domain and when domains are superimposed on C345C.

### C5 modulators alter C5 domain interfaces

Above we showed that when structures are superimposed on MG3, the C5a domain undergoes substantial displacement in most C5-modulator complexes while MG4 virtually retains its position. We therefore focused on these three domains to quantify how their rearrangement affects interdomain contacts. We measured changes in domain interface surface area between the MG3, MG4, and C5a domains and their neighbouring domains in C5-modulator complexes relative to apo C5 (**Table 2**), using PDBePISA (16).

**Table 2:** Domain interface surface area changes in C5-modulator complexes relative to apo C5.

C5-modulator complex	Δ domain interface surface area relative to apo C5 (Å²)												
	MG3 interface						MG4 interface			C5a interface			
	MG2	MG4	MG6	C5a	MG7	MG8	MG3	MG5	Link	MG3	MG6	C5d	MG8
C5-CVF	-176	137	-68	-321	267	41	137	-33	97	-321	-63	-24	-211
C5-CVF-SSL7	-169	154	-15	-268	313	64	154	-97	67	-268	-80	-11	-214
C5-CVF-H1H	-202	144	42	364	245	-109	144	-210	115	364	-136	-37	-151
C5-OmCI-RaCI1	-183	37	-193	-83	177	-250	37	186	-120	-83	-146	2	-150
C5-OmCI-RaCI2	-187	18	-189	-58	168	-264	18	177	-123	-58	-147	6	-188
C5-OmCI-RaCI3	-184	39	-198	-90	176	-259	39	172	-117	-90	-145	2	-144
C5-OmCI-RaCI1-CirpT1	-176	24	-88	-11	238	-184	24	133	-118	-11	-143	-35	-132
C5-Eculizumab	-168	47	-73	-355	319	-49	47	258	-120	-355	-153	8	-82
C5-K8	-174	16	-192	-850	314	-260	16	104	-134	-850	-278	26	-210
C5-K92	-196	-1	-119	-111	161	-194	-1	56	-134	-111	-140	-8	-113
C5-SSL7	3	26	53	-75	28	-6	26	-6	-38	-75	18	3	-72
C5-SSL7 β-grasp domain	-24	24	53	-31	58	-1	24	24	-39	-31	23	15	-72

Values represent the difference in interface surface area (Å²) between each C5-modulator complex and apo C5 for MG3, MG4, and C5a domains. Interface surface area was calculated as the total buried surface area between each domain and its neighbouring domains using PDBePISA (16). Positive values indicate an increase in interface area, while negative values indicate a decrease. Values are colour-coded from blue (decrease) to red (increase), with saturation proportional to the magnitude of change. The C5a interface values for C5-K8 should be viewed with caution, as the scissile loop was not resolved in the crystal structure due to high disorder.

Consistent with the domain alignment analysis, most C5-modulator complexes display pronounced changes in MG3 interfaces. Specifically, we found that the MG3/MG8 interface is decreased in most complexes, with the largest reductions in interface surface area observed in C5-OmCI-RaCI2 and C5-K8. By contrast, C5-CVF and C5-CVF-SSL7 show increased MG3/MG8 interface area, while C5-SSL7 and C5-SSL7 β-grasp domain show minimal changes. The MG3/MG7 interface is increased across all C5-modulator complexes, while the MG3/MG2 interface is reduced in all complexes except C5-SSL7. Similarly, the MG3/MG6 interface is reduced in most complexes except C5-SSL7, C5-SSL7 β-grasp domain, and C5-CVF-H1H. Notably, the MG3/MG4 interface shows small increases in nearly all complexes (+16 to +47 Å²) with larger increases in CVF-bound structures (+137 to +154 Å²). By contrast, the MG3/C5a interface is reduced in all complexes except C5-CVF-H1H.

We also found that the MG4 domain shows contrasting patterns of interface redistribution between CVF-bound and non-CVF complexes. In CVF-bound complexes, the MG4/linker interface surface area increases while the MG4/MG5 interface surface area decreases. Conversely, in most other complexes, the MG4/MG5 interface is increased while the MG4/linker interface is decreased. Notably, the C5-SSL7 and C5-SSL7 β-grasp domain show minimal changes in both interfaces. By contrast, the C5a domain shows reduced interface area with MG8 across all complexes, ranging from -72 Å² in C5-SSL7 β-grasp domain to -214 Å² in C5-CVF-SSL7. Similarly, the C5a/MG6 interface is reduced in most complexes except C5-SSL7 and C5-SSL7 β-grasp. Notably, the C5a/C5d interface shows minimal changes across all complexes.

### C5 modulators alter the conformation of and interactions within the C5a domain

**Figure 5:**
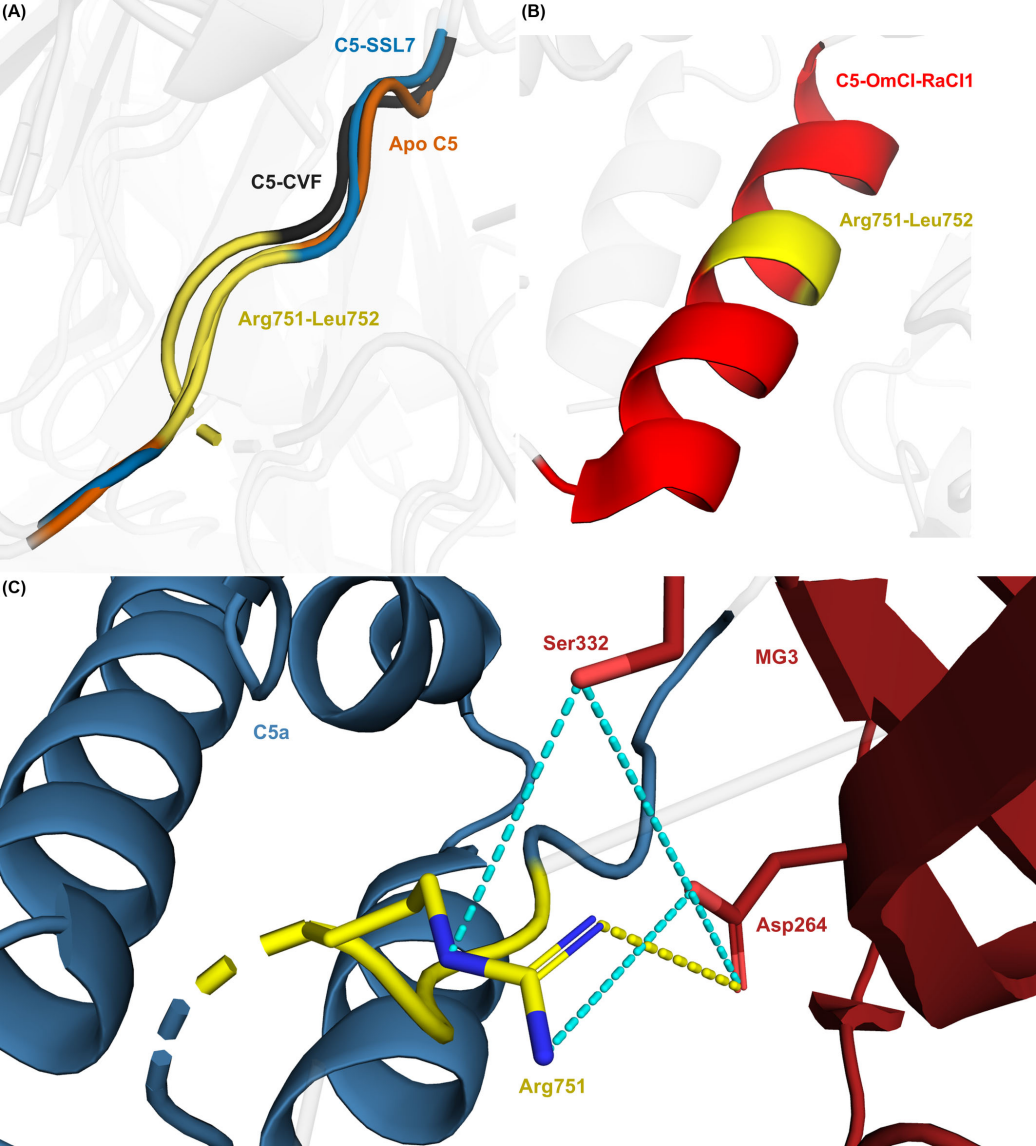
Scissile loop conformation in apo and selected C5-modulator complexes. **(A)** Superimposed structures of the C5 scissile loop in apo C5 (orange), C5-SSL7 (blue), and C5-CVF (dark grey) showing absence of an α-helical conformation. Arg751-Leu752 highlighted in yellow. **(B)** Structure of the scissile loop in C5-OmCI-RaCI1 (red), showing an α-helical conformation. Arg751-Leu752 highlighted in yellow. **(C)** Intramolecular interactions between C5a (steel blue) and MG3 (dark red) domains in C5-CVF, showing a salt bridge between Arg751 (yellow) and Asp264 (dark red) (dotted yellow line), and hydrogen bonds between Arg751, Ser332, and Asp264 (cyan dotted lines).

The C5a domain contains the scissile bond (Arg751-Leu752), the cleavage of which is essential for C5 activation. We showed that C5 modulators induce notable distance and rotational displacement of the C5a domain and alter its interface areas. To further characterise these effects, we analysed conformational changes within the C5a domain by examining the scissile loop (residues Asp746-Met754). In apo C5, this loop lacks any defined secondary structure (**Figure 5A**). Despite binding at distinct sites on C5, several modulators (C5-CVF-H1H, C5-OmCI-RaCI1/2/3, C5-OmCI-RaCI1-CirpT1, C5-Eculizumab, and C5-K92) induce the scissile loop to adopt an α-helical conformation (**Figure 5B**), confirmed by a Ramachandran plot analysis (**Supplementary Figure 1**). By contrast, the scissile loop remains unstructured in all other C5-modulator complexes.

To further investigate how C5 modulators affect the C5a domain, we analysed intramolecular bonds at the C5a/MG3 interface and observed the formation of hydrogen bonds between Arg751, Ser332, and Asp264, as well as a salt bridge between Arg751 and Asp264, in C5-CVF and C5-CVF-SSL7 (**Figure 5C**). By contrast, C5-CVF-H1H shows the same hydrogen bond (Arg751-Ser332) but lacks the salt bridge. Neither hydrogen bonds nor salt bridges involving Arg751 were observed in apo C5 or other C5-modulator complexes.

### C5-modulator complexes show altered dynamics and distinct rigidification patterns

**Figure 6:**
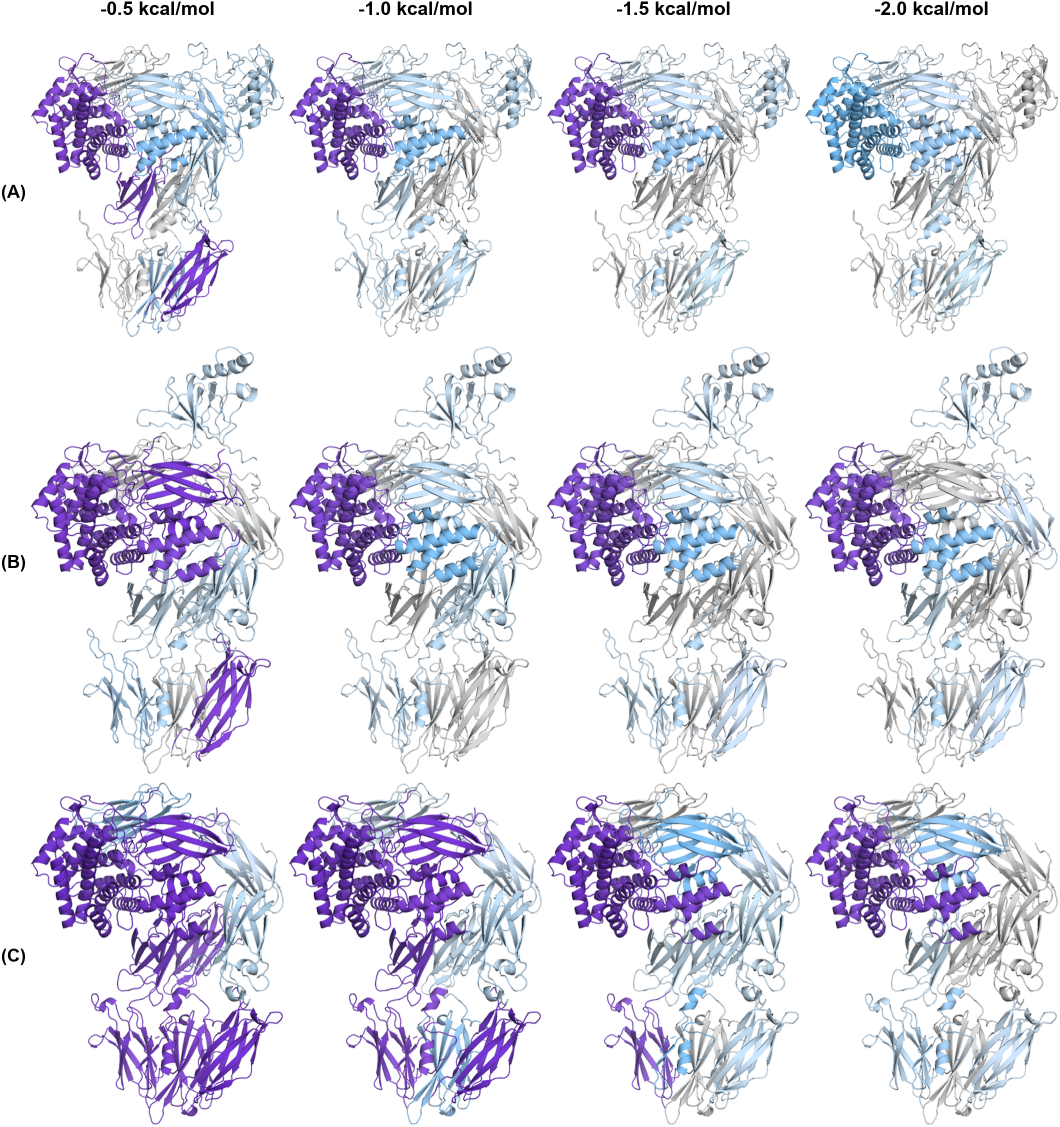
Rigid cluster decomposition of apo C5, C5-CVF, and C5-K8. **(A-C)** Rigid cluster decomposition (RCD) of **(A)** apo C5, **(B)** C5-CVF, and **(C)** C5-K8 at -0.5, -1.0, -1.5, and -2.0 kcal/mol energy cut-offs. Domains are coloured by the percentage of its Cα atoms assigned to one of the twenty largest rigid clusters: grey (0%), light blue (1-49%), and purple (≥50%), with colour intensity increasing proportionally within each range. RCD was conducted using the FLEXOME software suite (18).

**Figure 7:**
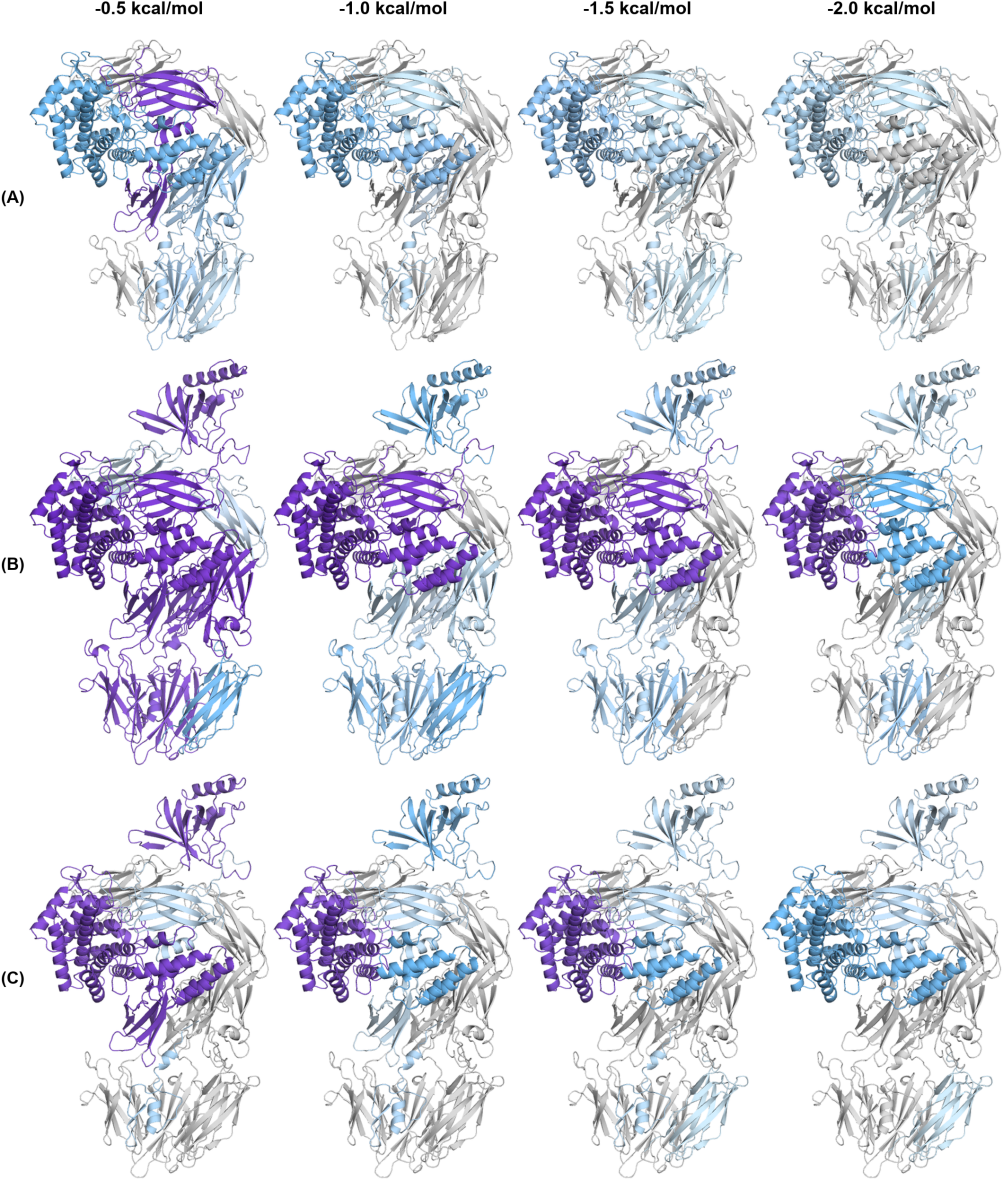
Rigid cluster decomposition of C5-CVF-H1H, C5-OmCI-RaCI1, and C5-OmCI-RaCI1-CirpT1. **(A-C)** Rigid cluster decomposition (RCD) of **(A)** C5-CVF-H1H, **(B)** C5-OmCI-RaCI1, and **(C)** C5-OmCI-RaCI1-CirpT1 at -0.5, -1.0, -1.5, and -2.0 kcal/mol energy cut-offs. Domains are coloured by the percentage of its Cα atoms assigned to one of the twenty largest rigid clusters: grey (0%), light blue (1-49%), and purple (≥50%), with colour intensity increasing proportionally within each range. RCD was conducted using the FLEXOME software suite (18).

Above we showed that modulators significantly alter the conformation of C5, inducing notable domain rearrangements and domain interface changes. We next analysed the dynamics and hydrogen bond architecture of C5 structures to determine how modulators redistribute structural constraints across C5. We performed a pebble-game rigidity analysis, using the FLEXOME software suite (18), to determine the size and retention of rigid clusters at -0.5, -1.0, -1.5, and -2.0 kcal/mol energy cut-offs across all C5 structures (**Figures 6**, **7**, **Supplementary Figures 2**-**8**). Specifically, rigidity was quantified as the percentage of domain Cα atoms assigned to one of the twenty largest rigid clusters for a given structure (**Supplementary Tables 13**-**16**). In this analysis, the four energy cut-offs represent a progressive stringency of the constraint network: at the least stringent cut-off (-0.5 kcal/mol), all hydrogen bonds identified by interatomic geometry contribute to the constraint network, whereas at increasingly negative cut-offs, hydrogen bonds below the chosen energy threshold are removed in order of increasing strength. Regions that remain rigid at more negative cut-offs are therefore supported by stronger constraint networks, while regions that are rigid only at less negative cut-offs depend on weaker hydrogen bonds. **Figures 6**, **7** each show three representative structures at the four cut-offs, with domains coloured by the percentage of Cα atoms assigned to the twenty largest rigid clusters. Reading from left to right within each panel thus reveals the order in which domains lose rigidity as the weakest bonds are progressively removed, and comparison across panels reveals how modulators redistribute these constraint networks relative to apo C5 (**Figure 6A**).

We observed that the C5d domain retains rigidity throughout the dilution series in nearly all structures (36-61% at -2.0 kcal/mol, excluding C5-CVF-H1H), providing an internal reference for cross-structure comparison. C5-CVF-H1H (**Figure 7A**) is a notable exception, where both C5d and C5a rigidity drop substantially at -2.0 kcal/mol (15% and 0%, respectively) compared to all other structures. By contrast, the MG8 domain shows the greatest variation across structures. Apo C5 (**Figure 6A**), C5-CVF-SSL7 (**Supplementary Figure 2**), and C5-SSL7 (**Supplementary Figure 7**) show consistently low MG8 rigidity (<10%) across all cut-offs, whereas C5-CVF (**Figure 6B**), C5-SSL7 β-grasp domain (**Supplementary Figure 8**), C5-Eculizumab (**Supplementary Figure 5**), and C5-CVF-H1H (**Figure 7A**) show elevated MG8 rigidity at -0.5 kcal/mol that drops below 10% by -1.0 kcal/mol. Meanwhile, C5-SSL7 and C5-SSL7 β-grasp domain differ substantially in MG8 rigidity at -0.5 kcal/mol (9% vs 63%) despite both containing the SSL7 β-grasp domain.

We also found that C5-K8 (**Figure 6C**), C5-K92 (**Supplementary Figure 6**), and the C5-OmCI-RaCI complexes (**Figure 7B**, **Supplementary Figures 3**, **4**) show the most extensive rigidity. In C5-K8, the largest rigid cluster at -0.5 kcal/mol spans nearly all domains except MG3 and MG7. Across all five complexes, MG8 remains elevated at -1.0 kcal/mol; at -2.0 kcal/mol, MG8 rigidity persists in C5-K8 (30%), C5-K92 (40%), and C5-OmCI-RaCI1 (49%) but drops to 0% in C5-OmCI-RaCI2 and C5-OmCI-RaCI3. C5a rigidity is also elevated in these complexes compared to apo C5, with C5-K8 showing the highest retention at -2.0 kcal/mol. MG1 shows elevated rigidity across these complexes compared to apo C5, where it is absent. C5-K92 differs from C5-K8 in showing higher CUB and MG7 rigidity. By contrast, C5-OmCI-RaCI complexes show elevated MG3 rigidity compared to C5-K8 and C5-K92. Within this group, MG5 uniquely maintains rigidity through -2.0 kcal/mol, which is not observed in other structures. C5-OmCI-RaCI3 shows elevated MG7 rigidity and the highest overall rigidity among the C5-OmCI-RaCI complexes, with MG2 retaining 70% at -2.0 kcal/mol. C5-OmCI-RaCI1-CirpT1 (**Figure 7C**) differs markedly from C5-OmCI-RaCI1, showing low MG8 rigidity across all cut-offs and reduced overall rigidity (25% vs 65% at -0.5 kcal/mol).

## Discussion

Complement component 5 (C5) occupies a central position in the complement cascade, and its cleavage into C5a and C5b by C5 convertases initiates potent inflammatory and cytolytic responses implicated in numerous autoimmune and inflammatory diseases. This has made it an attractive therapeutic target, with several inhibitors now in clinical use or development. Despite binding diverse sites across the C5 surface, these inhibitors achieve similar functional outcomes, inviting scrutiny about the structural basis for their efficacy. Our comparative analysis of C5-modulator complexes reveals that inhibitors induce conformational and dynamic changes extending well beyond their binding sites, supporting a significant role for allosteric mechanisms in C5 inhibition.

To understand how modulators alter C5 structure, we first examined the C5-CVF complex, which provides a structural template for convertase engagement. CVF functions as a C3b homologue that forms a stable fluid-phase convertase capable of cleaving C5 (5). While CVF is not identical to the physiological convertase, its complex with C5 provides the closest available structural approximation to the activated state of C5. Our integrated analysis reveals that CVF binding induces a distinctive pattern of structural changes that collectively appear to prime C5 for activation. When CVF engages C5 at the MG4/MG5 interface and the MG7 domain, the MG3 domain undergoes among the largest positional shifts observed across all complexes, accompanied by substantial repositioning of the CUB and C5d domains. At the level of domain interfaces, CVF binding produces a distinct interaction pattern shift: the MG4/linker interface expands while the MG4/MG5 interface contracts, and specific intramolecular contacts establish at the C5a/MG3 interface including the Arg751-Asp264 salt bridge that repositions the scissile bond residue. Furthermore, the MG3/MG8 interface increases in C5-CVF and C5-CVF-SSL7, a pattern that distinguishes these activator-bound structures from most inhibitor-bound complexes where this interface contracts. RCD analysis reveals that while C5-CVF shows elevated MG8 rigidity (percentage of domain Cα atoms assigned to one of the twenty largest rigid clusters) at permissive energy cut-offs, this constraint is lost at more stringent thresholds, and the overall rigidity remains comparable to apo C5. Together, this suggests that CVF-induced conformational changes reposition domains while preserving the flexibility that may be required for C5a release and C5b rearrangement. The CVF-bound state therefore provides a reference against which inhibitory mechanisms can be evaluated.

In contrast to CVF-induced activation, the tick-derived OmCI-RaCI and bovine knob domain K8 achieve potent inhibition through binding sites remote from the convertase interface. OmCI binds the C5d/CUB interface while RaCI variants bind the C5d/MG1/MG2 region; K8 binds MG8. Despite these disparate locations, our analysis reveals shared structural features that distinguish these complexes from the CVF-bound state. Most notably, the MG3/MG8 interface contracts substantially in all these complexes. These complexes also display extensive global rigidification: at permissive energy cut-offs, the largest rigid cluster spans nearly all domains, and elevated C5a rigidity persists through the dilution series. We observed compelling evidence for long-range allosteric communication in C5-K8: MG1 rigidity increases from negligible in apo C5 to substantial levels, comparable to the rigidification achieved by K92, which binds directly at the MG1/MG5 interface. This observation is broadly consistent with experimental HDX-MS studies (11) and suggests that the effects of K8 binding propagate across the entire C5 structure to constrain domains that never contact the modulator. Together, these observations suggest that these inhibitors trap C5 in a conformationally constrained state, though as discussed below, such rigidification is not universally required for inhibition.

The precision with which allosteric effects can be modulated is illustrated by comparing the three RaCI variants in complex with OmCI. Despite binding the same C5d/MG1/MG2 interface and achieving complete pathway inhibition, these related proteins establish measurably different constraint networks across C5. RaCI3 generates the most extensively rigidified complex and uniquely maintains substantial MG2 rigidity at the most stringent energy cut-off, indicating a stronger local hydrogen bond network than observed for the C5-OmCI-RaCI1 or C5-OmCI-RaCI2. This difference extends beyond the immediate binding site: RaCI3 also induces elevated MG7 rigidity not observed with the other variants. These findings suggest that allosteric inhibition is not a binary state but rather a tunable property, where subtle differences in binding interactions translate into distinct patterns of mechanical constraint. The functional significance of this tunability remains to be determined, but it implies that rational optimisation of allosteric inhibitors could achieve precise control over the distribution of structural constraints across C5.

Central to understanding how binding events at disparate sites produce widespread structural effects is the behaviour of the MG3 domain, which emerges from our analysis as a critical node in the conformational dynamics of C5. The concept of a “conformational relay” is instructive here: a domain that transmits perturbations from one region to another must itself be capable of movement and must couple productively to its neighbours. MG3 displays precisely these properties, undergoing large positional shifts while maintaining low rigidity, and forming interfaces with both the superhelix (MG7) and the C5a domain that respond systematically to modulator binding. The MG3/MG7 interface increases universally across all modulator complexes, suggesting enhanced mechanical coupling upon any perturbation of the C5 scaffold. Conversely, the MG3/C5a interface is reduced in nearly all inhibitor-bound structures. The C5-OmCI-RaCI complexes present an interesting variation: here MG3 displays elevated rigidity, potentially reflecting constraints imposed by RaCI binding at adjacent domains. This suggests that the relay function of MG3 can itself be modulated, either facilitated by maintaining flexibility or augmented by rigidification that locks the domain into a displaced position. The central role of MG3 in propagating conformational signals makes it an attractive target for therapeutic intervention, though the complexity of its behaviour across different inhibitor contexts cautions against simplistic targeting strategies.

The comparison between K92 and K8 reveals that the structural consequences of modulator binding are determined by the molecular interactions established, not by binding site alone. Both are bovine knob domains that achieve partial pathway inhibition and high global rigidity, but with markedly different structural properties. K8 binds MG8 and induces major allosteric rigidification, while keeping the scissile loop unstructured. By contrast, K92 binds the MG1/MG5 interface, the same site as SSL7, yet induces scissile loop helicalisation, which is absent in SSL7. Similarly, eculizumab binds MG7 and achieves complete inhibition of both pathways with only moderate rigidity. These comparisons argue against a simple model in which binding sites are classified as “allosteric” or “orthosteric” based on location; rather, the nature of molecular recognition at each site determines the downstream structural and dynamic consequences.

The scissile loop provides the most direct structural readout of inhibitor impact on the cleavage site. Structurally unrelated inhibitors binding across the C5 surface, including OmCI-RaCI (with or without CirpT1), eculizumab, K92, and H1H, all induce α-helical restructuring of this normally unstructured region. Jore et al. (8) proposed that a priming event is required for C5 activation, and helicalisation may represent a structural barrier to this priming, locking the region into a conformation incompatible with productive convertase engagement. However, scissile loop helicalisation is neither necessary nor sufficient for complete inhibition. SSL7 achieves complete AP inhibition without inducing helicalisation (10), while K92 induces helicalisation but fails to completely inhibit either pathway. The most effective inhibitors in our dataset, including eculizumab and OmCI-RaCI, combine scissile loop helicalisation with other structural features such as interface remodelling and appropriate levels of rigidification. This suggests that helicalisation contributes to but does not guarantee inhibitory efficacy.

Whether inhibitors that contact the convertase-binding surface function through simple steric blockade or also induce conformational changes is understudied. Eculizumab binds MG7, directly contacting the convertase interface, and was proposed to function through steric occlusion (4). However, we observed that eculizumab induces substantial MG3 displacement, scissile loop helicalisation, and the largest increase in MG3/MG7 interface area among all complexes, features characteristic of conformational modulation beyond simple occlusion of convertase binding. The most informative test case is the comparison between C5-OmCI-RaCI1 and C5-OmCI-RaCI1-CirpT1. CirpT1 binds the MG4 domain, directly overlapping the CVF-binding surface, and was proposed to inhibit through steric blocking of convertase docking (9). The addition of CirpT1 to the OmCI-RaCI1 complex dramatically reduces overall rigidity at -0.5 kcal/mol from 65% to 25%, eliminates the elevated MG8 rigidity characteristic of the parent complex, and reduces MG3 rigidity to negligible levels. However, complete inhibition is maintained. This demonstrates that steric occlusion is sufficient for inhibition even when allosteric rigidification is substantially relaxed, and that steric and allosteric mechanisms can produce antagonistic rather than additive effects on the conformational landscape. This has direct implications for combination therapy design, where inhibitor pairs must be evaluated for their integrated effects on C5 dynamics.

The SSL7 complexes provide the clearest example of inhibition achieved without conformational perturbation. SSL7 binds the MG1/MG5 interface and achieves complete AP inhibition with only partial CP inhibition, yet the C5 structure remains essentially native: domain displacement is minimal, interface changes are negligible, the scissile loop remains unstructured, and global rigidity resembles that of apo C5. Laursen et al. (10) proposed that SSL7 functions through IgA-dependent steric hindrance, with the β-grasp domain anchoring to C5 while the OB domain recruits IgA to create a physical barrier. Our data strongly support this model and demonstrate that the isolated β-grasp domain contributes no allosteric component to inhibition despite sharing the binding site with K92; its sole function appears to be molecular positioning. The incomplete CP inhibition can thus be understood to be a consequence of the following minimal mechanism: without conformational modulation, inhibition depends entirely on the steric barrier provided by recruited IgA, which may be incomplete for certain convertase geometries. Future structural studies of the complete SSL7-IgA-C5 complex would be valuable to test this model directly.

The C5-CVF-H1H complex occupies a unique position in our dataset that illuminates the conformational trajectory of C5 activation. This structure combines features of CVF-induced priming with characteristics that distinguish it from both fully activated and fully inhibited states. The MG3/C5a interface is substantially increased, opposite to the pattern in inhibitor-bound structures, suggesting that CVF-induced anchoring of C5a to the superhelix core is preserved. The scissile loop adopts an α-helical conformation, and the Arg751-Ser332 hydrogen bond characteristic of CVF-bound structures is present. However, the Arg751-Asp264 salt bridge is absent, representing incomplete formation of the activating contacts observed in C5-CVF and C5-CVF-SSL7. The MG3/MG8 interface is decreased, indicating that H1H binding at the C5a/MG8 interface disrupts this coupling. Most distinctively, global rigidity is the lowest observed in any complex at stringent energy cut-offs, with both C5d and C5a losing constraint density. This constellation of features suggests that C5-CVF-H1H represents a state in which CVF has induced conformational priming but H1H binding prevents completion of the activation trajectory. The unique structural signature of this complex provides valuable insights into which features of CVF-induced priming are essential for activation and which can be disrupted while maintaining an overall primed-like conformation.

The question of why some inhibitors achieve complete pathway blockade while others achieve only partial inhibition is essential to understanding the structural requirements for effective C5 therapeutics. K8, K92, and SSL7 each fail to achieve complete inhibition of both pathways, despite having entirely different structural profiles: C5-K8 shows high overall rigidity but lacks scissile loop restructuring; C5-K92 achieves comparable rigidification with scissile loop helicalisation but produces relatively modest domain displacement; C5-SSL7 maintains an essentially native C5 conformation. The absence of a shared structural deficit among partial inhibitors indicates that complete inhibition cannot be achieved by maximising any single structural parameter. Complete inhibitors such as eculizumab and the OmCI-RaCI complexes achieve efficacy through different structural combinations. This diversity suggests that effective inhibition requires perturbation across multiple structural parameters, and that deficiency in different parameters can independently result in incomplete inhibition. From an evolutionary perspective, this may explain why certain pathogens have evolved inhibitors to bind at multiple sites of C5 simultaneously: combinatorial targeting may more reliably achieve the multifactorial perturbation required for complete inhibition.

Beyond C5, a comparable pattern of redundant targeting by mechanistically and structurally distinct inhibitors has been documented at multiple nodes of the complement cascade. The classical pathway initiating proteases C1r and C1s are independently targeted by *Borrelia burgdorferi* BBK32, which traps C1r in its zymogen form within the C1 complex (23), and by gigastasin from the giant Amazon leech, which engages the active site and exosite of C1s and MASP-2 (24). Likewise, factor H is recruited to the surface of *B. burgdorferi* by a family of complement regulator-acquiring surface proteins (CRASPs) with distinct binding profiles (25), and to *Neisseria meningitidis* by the unrelated lipoprotein fHbp (26). *S. aureus* targets C3 and its convertases through at least three mechanistically distinct families, SCIN, Efb/Ehp, and Sbi, that variously trap the assembled convertase, allosterically impair convertase formation, or sequester C3 fragments (27, 28). The recurrence of this strategy across unrelated pathogens and at different points in the cascade suggests that what we propose for C5 may reflect a more general evolutionary solution: where single-site engagement of a large, allosterically networked complement component is insufficient to robustly suppress its function, redundant or combinatorial targeting provides a more reliable route to functional blockade.

Amid the conformational variability induced by different modulators, certain structural relationships remain invariant. The stability of the C5a/C5d interface across all complexes suggests this interaction surface represents an essential architectural feature that cannot be perturbed without compromising structural integrity. This interface may function as a fixed pivot point around which other interdomain relationships reorganise. Similarly, the superdomain comprising CUB, C5d, and MG8 maintains its internal geometry across all structures, moving as a rigid unit when displaced. The greater rigidity retention of the C5d domain across most structures suggests that C5d harbours the rigid core of C5, analogous to the folding cores identified through rigidity dilution in other proteins (29). The universality of certain interface changes is likewise informative: the MG3/MG7 interface increases in every complex without exception, while the C5a/MG8 interface decreases in every complex. These invariant responses suggest that any perturbation of the C5 scaffold channels through common structural pathways. Understanding these invariant relationships may be as important for therapeutic design as understanding the differences between inhibitors, as they define the constraints within which any modulator must operate.

Collectively, these findings reframe our understanding of C5 inhibition from a binding-site-centric view to a mechanism-centric view. The convergence of diverse inhibitors on scissile loop helicalisation suggests this conformational state may represent a druggable endpoint achievable from multiple surface locations, though helicalisation alone does not guarantee complete inhibition. The identification of MG3 as a conformational relay and MG8 as a domain whose rigidity reports on global constraint status highlights potential nodes in the allosteric network. However, the partial inhibition achieved by K8 despite direct MG8 binding cautions that targeting individual nodes may be insufficient. The demonstration that steric and allosteric mechanisms are independent, and can antagonise each other when combined, provides insights for rational combination therapy design.

Several limitations warrant consideration. The structures examined in this study were determined by X-ray crystallography and cryo-electron microscopy at resolutions ranging from 2.3 Å to 4.3 Å, capturing static snapshots that may not fully represent the conformational ensemble sampled in solution. Crystal packing contacts, which vary between structures, may also influence domain positions in ways that do not reflect solution behaviour. The C345C domain could not be resolved in several structures and displays extreme positional variability where present, likely reflecting crystallographic disorder rather than functionally meaningful results. Hydrogen bond detection in the RCD analysis is resolution-dependent, which could affect comparisons between structures determined at lower resolution. RCD assumes a specific hydrogen bond energy model that, while well-validated, may not capture all mechanically relevant features. Furthermore, our analysis focuses on structural and dynamic properties without directly measuring functional outcomes such as convertase binding affinity or cleavage rates. The absence of a true C5-convertase structure limits our ability to definitively characterise the activated state, and our reliance on C5-CVF as a proxy should be considered when interpreting activation-related claims. Future studies employing molecular dynamics simulations, hydrogen-deuterium exchange mass spectrometry, or single-molecule techniques could provide complementary perspectives on the dynamic behaviour of C5-modulator complexes.

In conclusion, our structural analysis reveals that C5 inhibitors operating through diverse mechanisms induce characteristic patterns of conformational and dynamic change that distinguish activation-competent from activation-resistant states. The CVF-bound structure provides a reference for convertase engagement, characterised by MG3 repositioning, expansion of the MG4/linker interface with concomitant MG4/MG5 contraction, formation of specific C5a-MG3 interactions, and increased MG3/MG8 interface area. Predominantly allosteric inhibitors, such as the OmCI-RaCI complexes, invert key features of this pattern, contracting the MG3/MG8 interface, restructuring the scissile loop in most cases, and imposing global rigidification. Predominantly steric inhibitors can achieve inhibition through direct occlusion without requiring allosteric rigidification, as observed with SSL7 for example. Notably, complete inhibitors can achieve efficacy through different structural combinations, indicating that multiple routes to effective inhibition exist. This is well-demonstrated by eculizumab, which binds the CVF-shared MG7 site but also induces scissile loop helicalisation. Taken together, the insights presented in this paper provide a structural basis for understanding complement C5 regulation and may guide the rational design of next-generation therapeutics for complement-mediated diseases.

## Data Availability

All structures analysed are publicly available from the Protein Data Bank; accession codes are listed in **Table 1**. Custom code for domain displacement analysis is deposited in Zenodo (https://doi.org/10.5281/zenodo.18622373). All other data are available from the corresponding authors upon reasonable request.
