# Supplementary figures and images for: Modulator-induced conformational changes in complement C5, implications for function and drug design
Source: Front Immunol. 2026 May 15;17:1834455. doi: 10.3389/fimmu.2026.1834455 (PMC13219244; doi:10.3389/fimmu.2026.1834455)

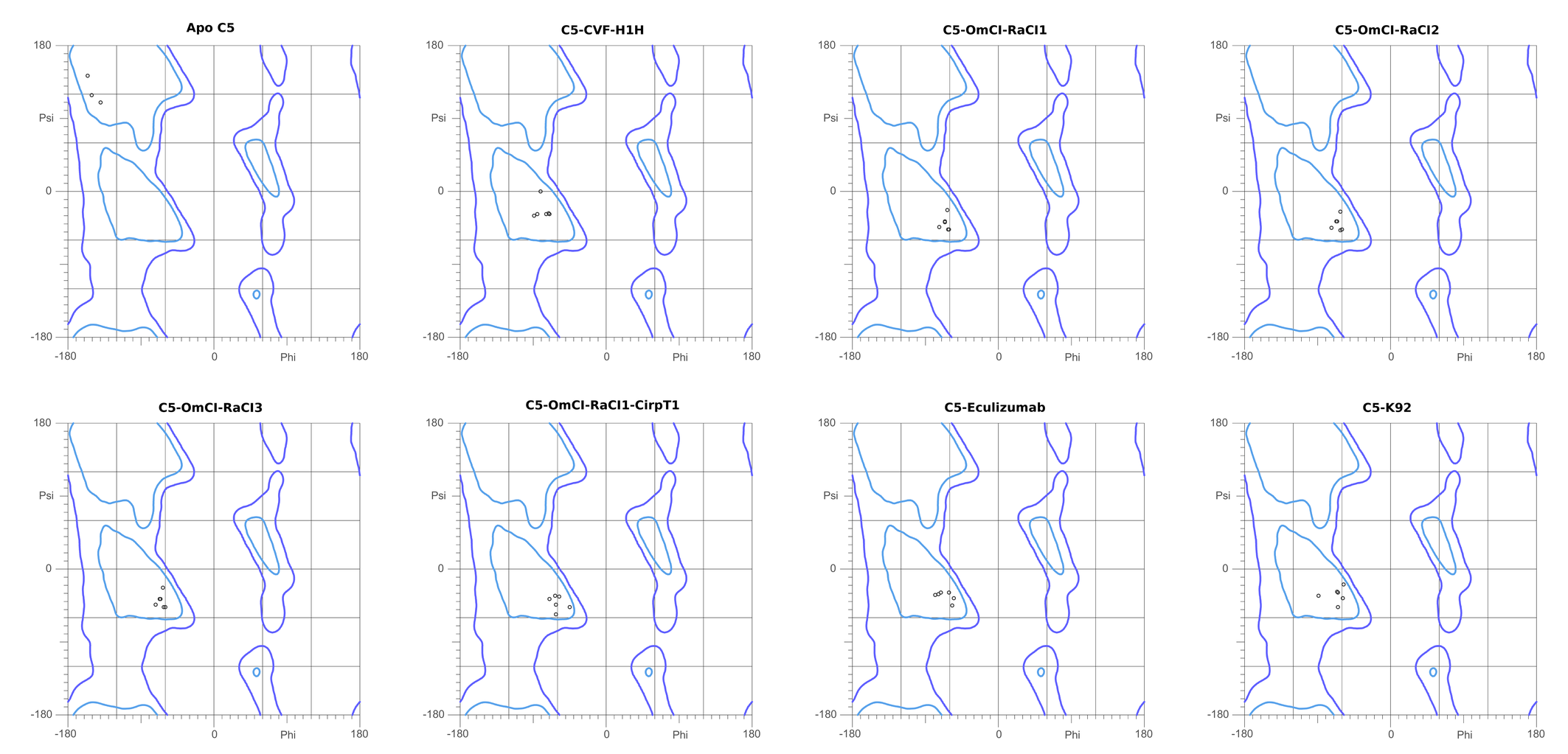

Supplement: Supplementary file 1 [file Image1.tif]

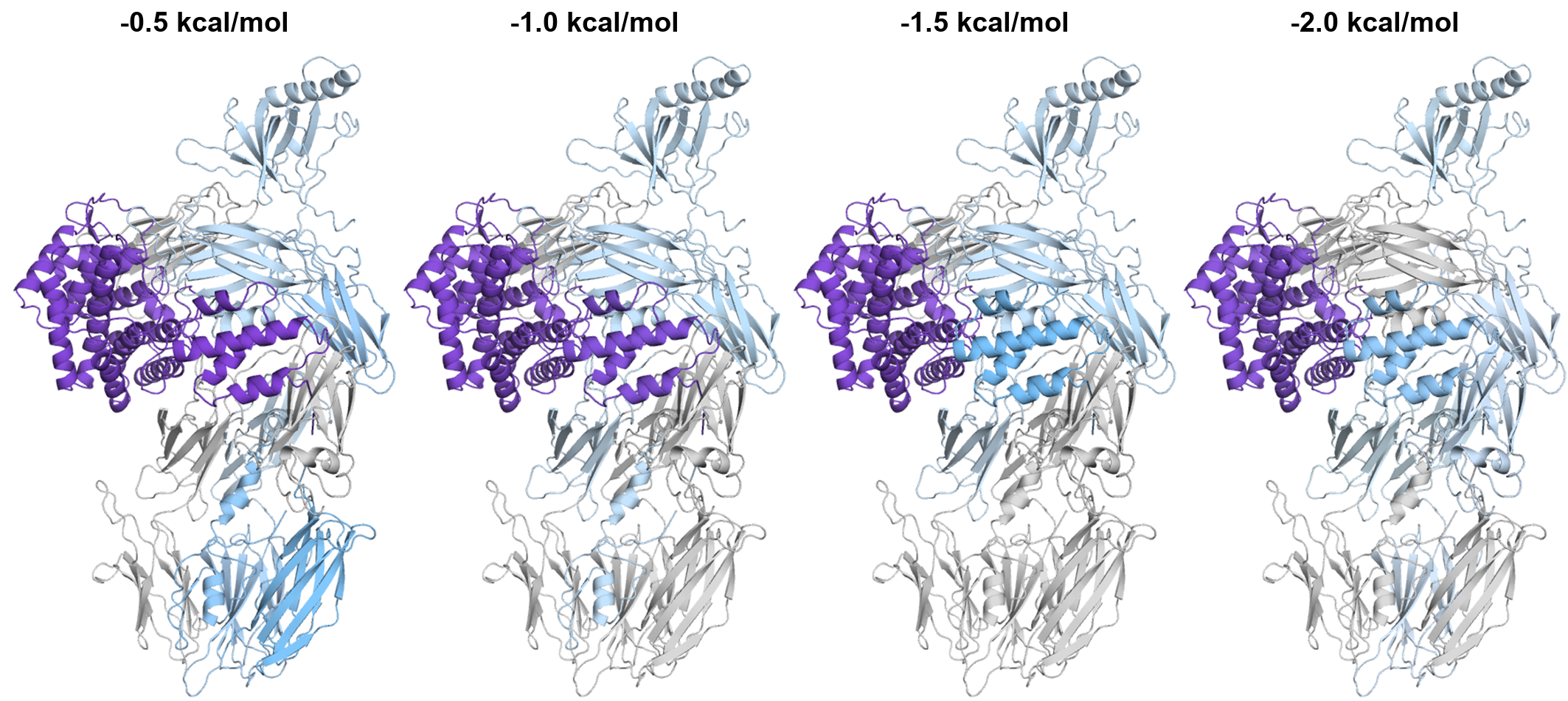

Supplement: Supplementary file 2 [file Image2.tif]

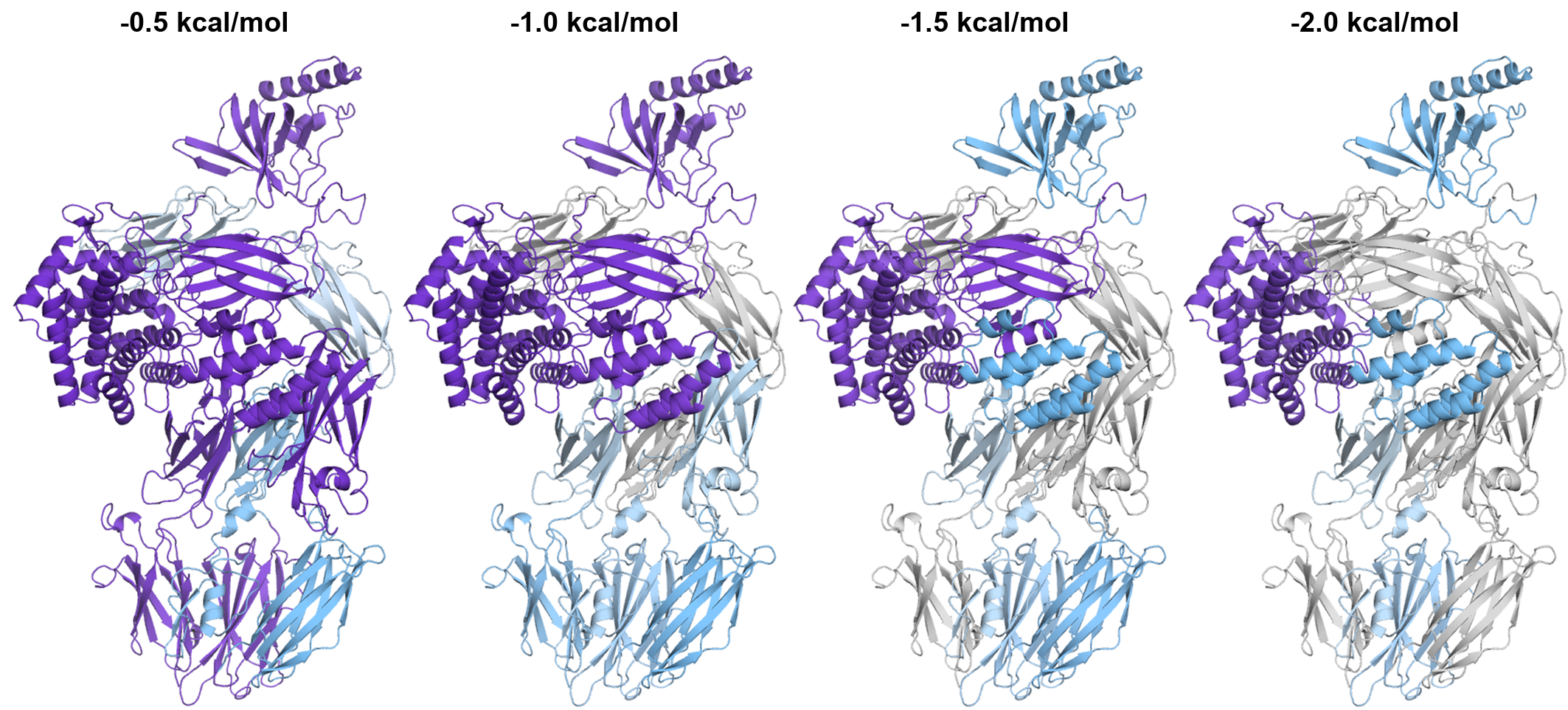

Supplement: Supplementary file 3 [file Image3.tif]

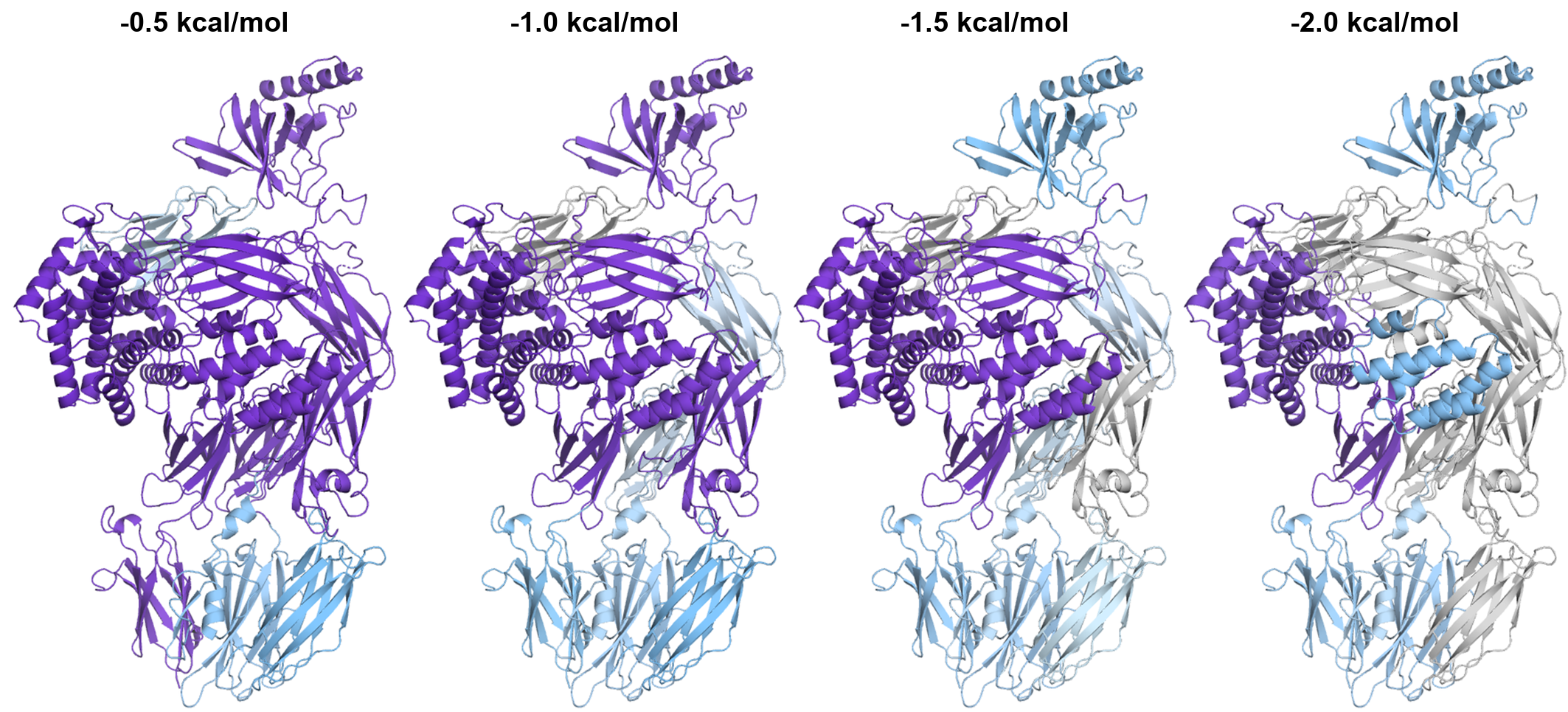

Supplement: Supplementary file 4 [file Image4.tif]

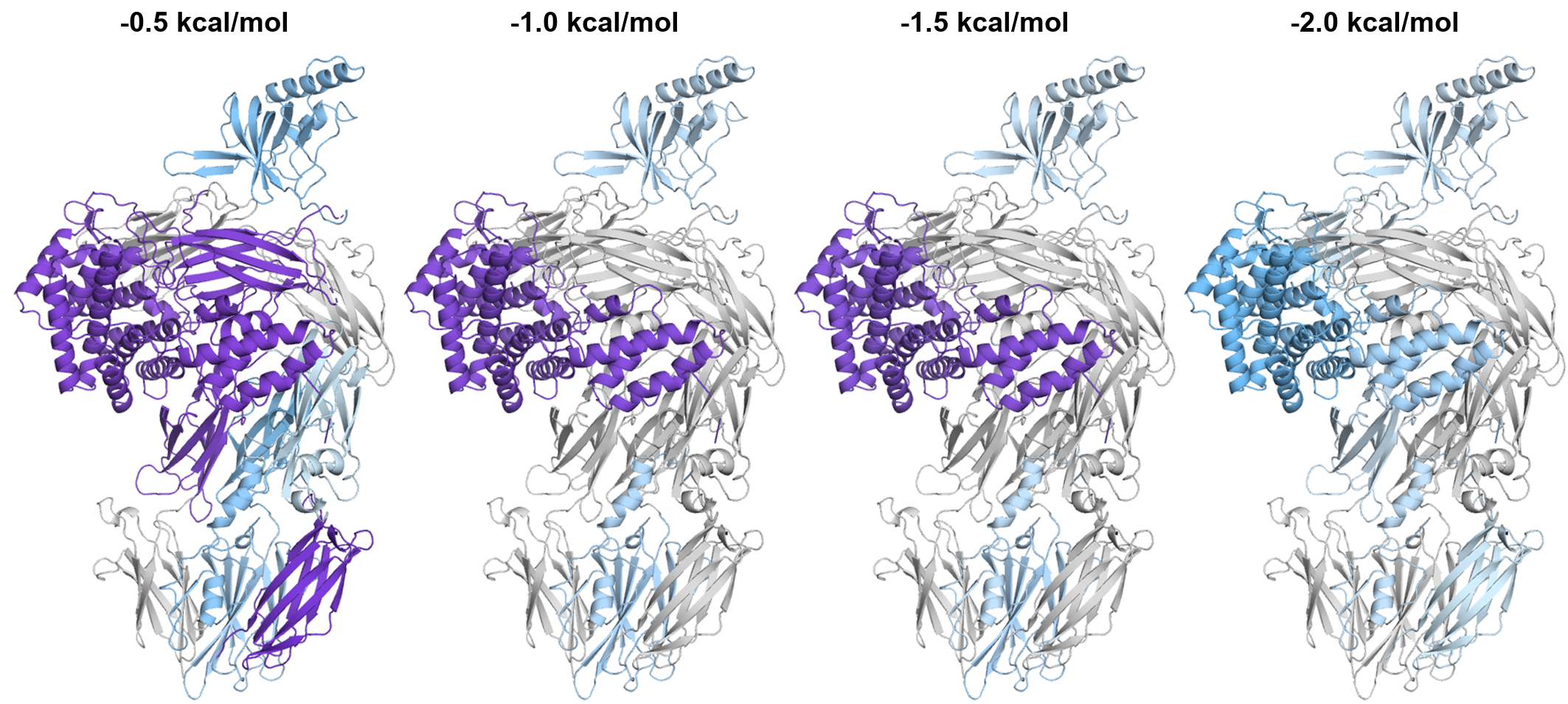

Supplement: Supplementary file 5 [file Image5.tif]

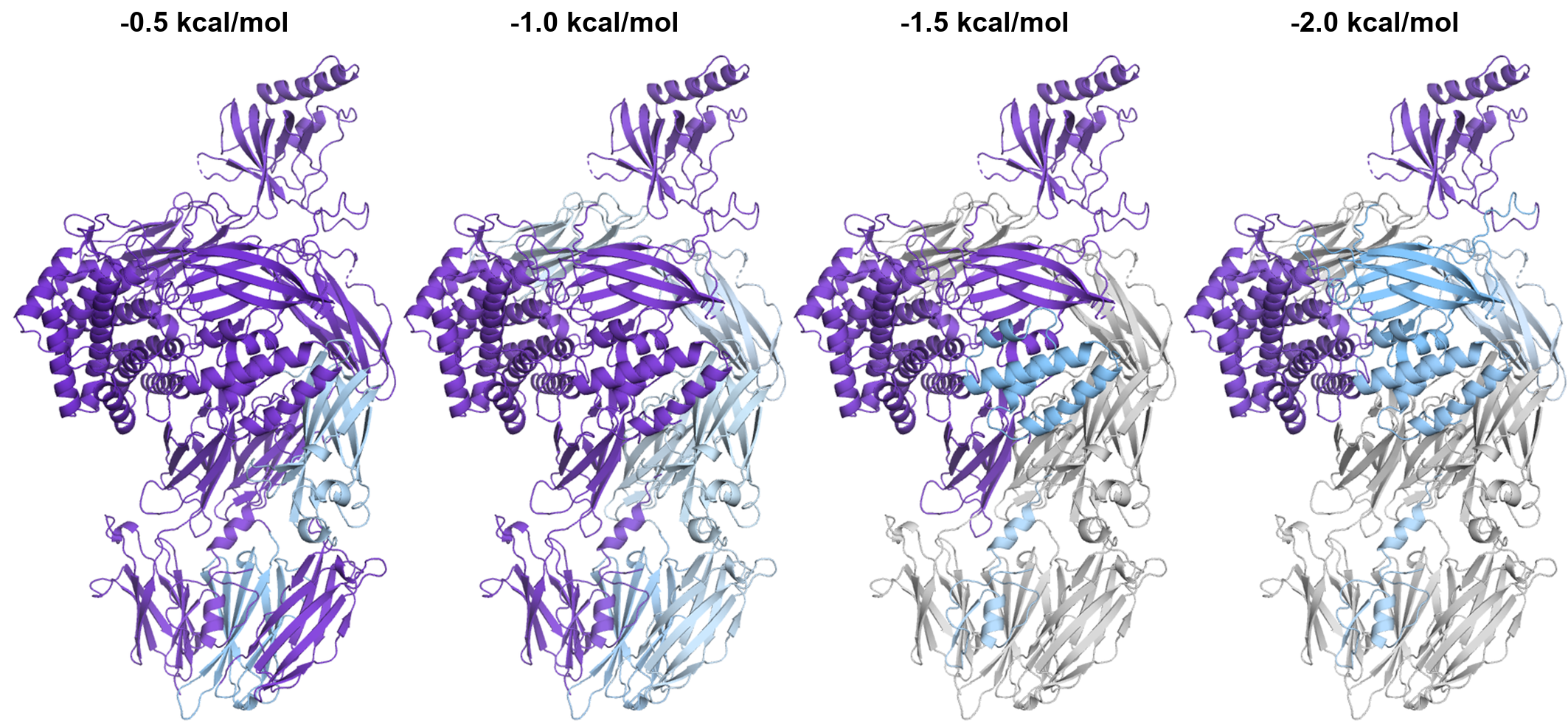

Supplement: Supplementary file 6 [file Image6.tif]

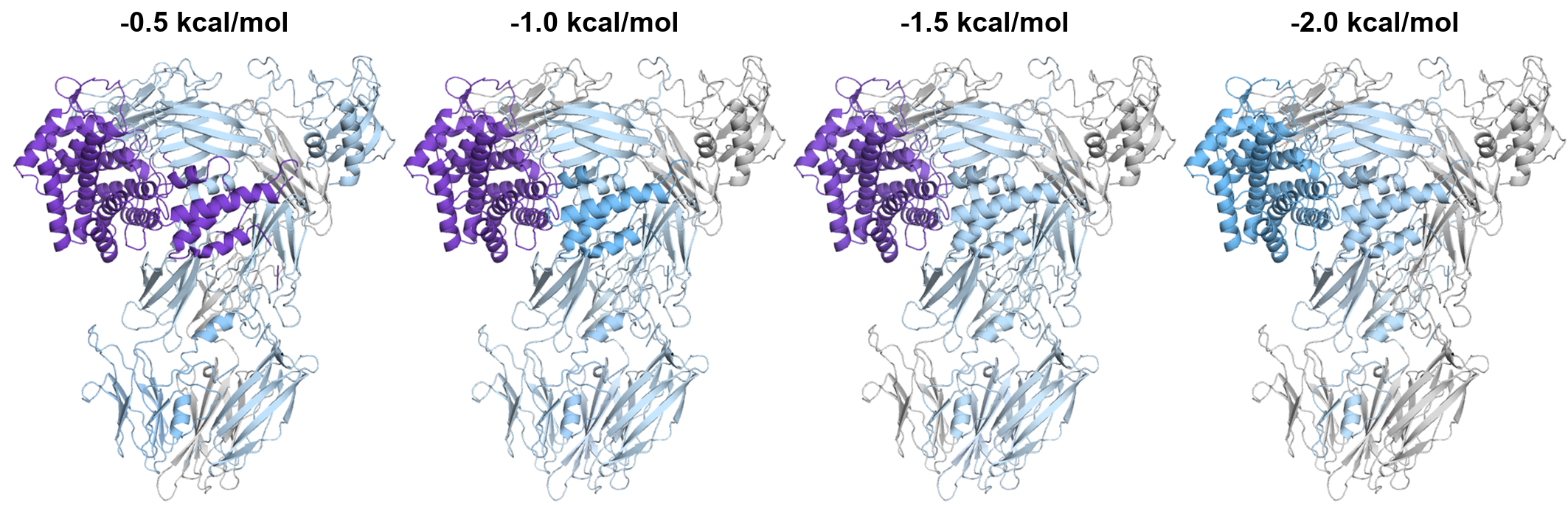

Supplement: Supplementary file 7 [file Image7.tif]

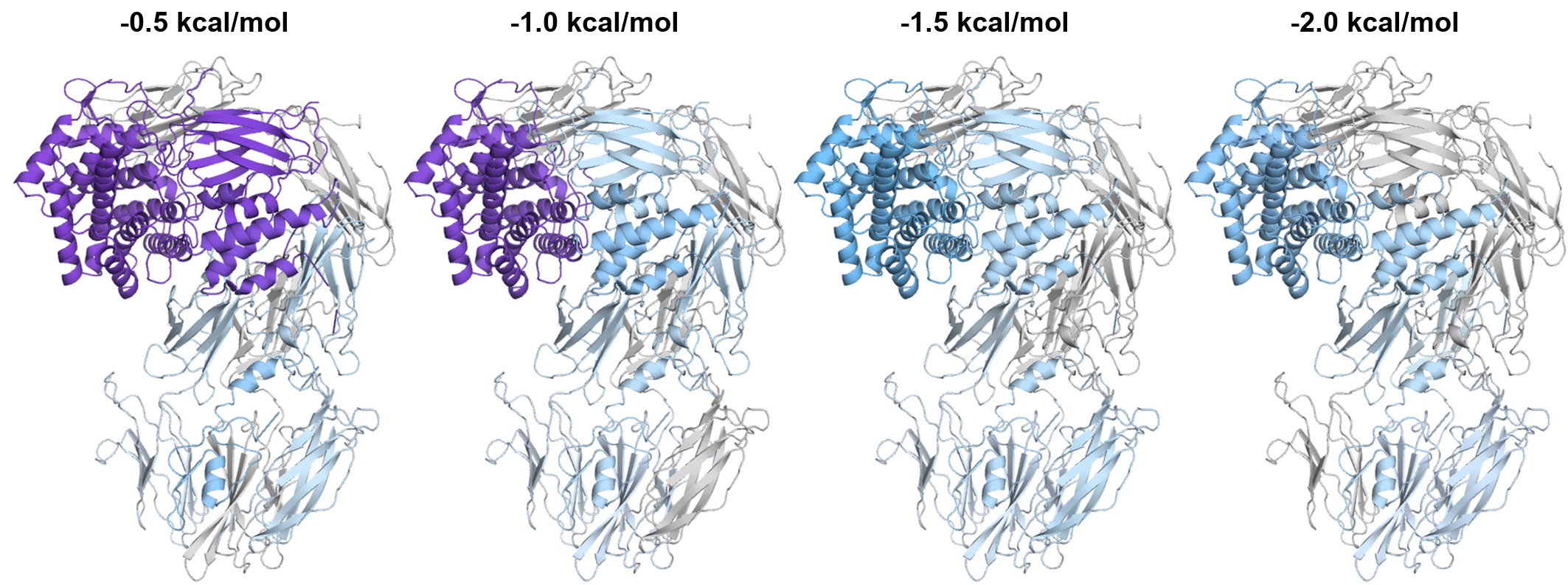

Supplement: Supplementary file 8 [file Image8.tif]
