# Supplementary material for: Modulator-induced conformational changes in complement C5, implications for function and drug design
Source: Front Immunol. 2026 May 15;17:1834455. doi: 10.3389/fimmu.2026.1834455 (PMC13219244; doi:10.3389/fimmu.2026.1834455)
Supplement: Supplementary file 9 [file Table1.docx]

**Supplementary Figure 1. Ramachandran plot of the C5a domain scissile loop residues.** Phi (φ) and psi (ψ) angles for residues Asp746-Met754 in apo C5 and selected C5-modulator complexes. Blue contours represent favoured (inner) and allowed (outer) regions for general amino acid residues. In apo C5, resolved scissile loop residues adopt extended backbone conformations in the β-region without defined secondary structure. In the depicted C5-modulator complexes, residues cluster within the right-handed α-helix region, indicating α-helical structure. Ramachandran plots were generated using MolProbity (17).

**Supplementary Figure 2. Rigid cluster decomposition of C5-CVF-SSL7.** Rigid cluster decomposition (RCD) of C5-CVF-SSL7 at -0.5, -1.0, -1.5, and -2.0 kcal/mol energy cut-offs. Domains are coloured by the percentage of its Cα atoms assigned to one of the twenty largest rigid clusters: grey (0%), light blue (1-49%), and purple (≥50%), with colour intensity increasing proportionally within each range. RCD was conducted using the FLEXOME software suite (18).

**Supplementary Figure 3. Rigid cluster decomposition of C5-OmCI-RaCI2.** Rigid cluster decomposition (RCD) of C5-OmCI-RaCI2 at -0.5, -1.0, -1.5, and -2.0 kcal/mol energy cut-offs. Domains are coloured by the percentage of its Cα atoms assigned to one of the twenty largest rigid clusters: grey (0%), light blue (1-49%), and purple (≥50%), with colour intensity increasing proportionally within each range. RCD was conducted using the FLEXOME software suite (18).

**Supplementary Figure 4. Rigid cluster decomposition of C5-OmCI-RaCI3.** Rigid cluster decomposition (RCD) of C5-OmCI-RaCI3 at -0.5, -1.0, -1.5, and -2.0 kcal/mol energy cut-offs. Domains are coloured by the percentage of its Cα atoms assigned to one of the twenty largest rigid clusters: grey (0%), light blue (1-49%), and purple (≥50%), with colour intensity increasing proportionally within each range. RCD was conducted using the FLEXOME software suite (18).

**Supplementary Figure 5. Rigid cluster decomposition of C5-Eculizumab.** Rigid cluster decomposition (RCD) of C5-Eculizumab at -0.5, -1.0, -1.5, and -2.0 kcal/mol energy cut-offs. Domains are coloured by the percentage of its Cα atoms assigned to one of the twenty largest rigid clusters: grey (0%), light blue (1-49%), and purple (≥50%), with colour intensity increasing proportionally within each range. RCD was conducted using the FLEXOME software suite (18).

**Supplementary Figure 6. Rigid cluster decomposition of C5-K92.** Rigid cluster decomposition (RCD) of C5-K92 at -0.5, -1.0, -1.5, and -2.0 kcal/mol energy cut-offs. Domains are coloured by the percentage of its Cα atoms assigned to one of the twenty largest rigid clusters: grey (0%), light blue (1-49%), and purple (≥50%), with colour intensity increasing proportionally within each range. RCD was conducted using the FLEXOME software suite (18).

**Supplementary Figure 7. Rigid cluster decomposition of C5-SSL7.** Rigid cluster decomposition (RCD) of C5-SSL7 at -0.5, -1.0, -1.5, and -2.0 kcal/mol energy cut-offs. Domains are coloured by the percentage of its Cα atoms assigned to one of the twenty largest rigid clusters: grey (0%), light blue (1-49%), and purple (≥50%), with colour intensity increasing proportionally within each range. RCD was conducted using the FLEXOME software suite (18).

**Supplementary Figure 8. Rigid cluster decomposition of C5-SSL7 β-grasp domain.** Rigid cluster decomposition (RCD) of C5-SSL7 β-grasp domain at -0.5, -1.0, -1.5, and -2.0 kcal/mol energy cut-offs. Domains are coloured by the percentage of its Cα atoms assigned to one of the twenty largest rigid clusters: grey (0%), light blue (1-49%), and purple (≥50%), with colour intensity increasing proportionally within each range. RCD was conducted using the FLEXOME software suite (18).

**Supplementary Table 1. Domain displacement in C5-CVF complex relative to apo C5**. Centre-of-mass translational (Å) and rotational (°) displacement when each C5-CVF domain is superimposed onto each apo C5 domain. The Cα root-mean-square deviation (RMSD) for each domain superimposition is shown below.

| **Complex** | **Metric** | **Apo C5** | | | | | | | | | | | | |
| --- | --- | --- | --- | --- | --- | --- | --- | --- | --- | --- | --- | --- | --- | --- |
|  |  | **MG1** | **MG2** | **MG3** | **MG4** | **MG5** | **MG6** | **Linker** | **C5a** | **MG7** | **CUB** | **C5d** | **MG8** | **C345C** |
| **MG1** | Distance (Å) | - | 0.8 | 22.6 | 4.5 | 0.7 | 2.0 | 2.7 | 12.8 | 15.1 | 6.4 | 9.0 | 6.0 | 111.8 |
| **MG1** | Angle (°) | - | 0.9 | 20.8 | 6.2 | 1.2 | 2.4 | 6.9 | 9.8 | 10.9 | 4.4 | 7.6 | 4.0 | 64.2 |
| **MG2** | Distance (Å) | 2.6 | - | 14.1 | 11.3 | 4.0 | 0.3 | 7.5 | 6.6 | 7.3 | 2.9 | 3.2 | 2.2 | 75.7 |
| **MG2** | Angle (°) | 3.9 | - | 22.3 | 14.5 | 5.2 | 0.7 | 14.3 | 8.5 | 9.3 | 3.0 | 5.0 | 2.5 | 65.7 |
| **MG3** | Distance (Å) | 5.9 | 3.7 | - | 6.9 | 4.1 | 3.1 | 3.0 | 3.4 | 6.1 | 3.0 | 2.9 | 2.8 | 102.6 |
| **MG3** | Angle (°) | 5.2 | 4.4 | - | 10.0 | 4.1 | 4.9 | 3.9 | 7.4 | 8.7 | 2.0 | 1.9 | 0.8 | 116.3 |
| **MG4** | Distance (Å) | 0.7 | 5.0 | 8.5 | - | 0.6 | 4.6 | 2.8 | 15.0 | 15.4 | 6.8 | 10.1 | 8.0 | 147.2 |
| **MG4** | Angle (°) | 0.9 | 6.4 | 12.3 | - | 0.2 | 5.1 | 7.1 | 14.6 | 12.4 | 4.1 | 7.5 | 6.1 | 102.4 |
| **MG5** | Distance (Å) | 2.0 | 3.7 | 17.6 | 2.5 | - | 2.7 | 1.6 | 17.5 | 17.8 | 8.5 | 11.9 | 9.0 | 127.2 |
| **MG5** | Angle (°) | 4.6 | 4.9 | 18.1 | 5.6 | - | 2.7 | 5.3 | 13.6 | 13.1 | 5.0 | 8.7 | 6.2 | 76.0 |
| **MG6** | Distance (Å) | 4.4 | 0.2 | 12.9 | 11.8 | 5.4 | - | 8.8 | 9.3 | 6.0 | 1.8 | 3.4 | 1.8 | 59.7 |
| **MG6** | Angle (°) | 5.5 | 0.4 | 20.9 | 15.1 | 6.8 | - | 13.9 | 10.3 | 9.6 | 0.3 | 3.9 | 2.0 | 55.3 |
| **Linker** | Distance (Å) | 1.4 | 1.6 | 14.0 | 2.5 | 1.1 | 2.0 | - | 11.4 | 12.9 | 4.8 | 7.4 | 5.1 | 121.2 |
| **Linker** | Angle (°) | 3.2 | 3.0 | 18.4 | 6.3 | 3.7 | 3.1 | - | 11.1 | 10.8 | 3.4 | 6.6 | 4.0 | 80.7 |
| **C5a** | Distance (Å) | 4.3 | 3.6 | 9.8 | 14.5 | 6.2 | 3.8 | 9.2 | - | 6.4 | 2.7 | 2.8 | 2.2 | 108.3 |
| **C5a** | Angle (°) | 2.9 | 4.4 | 22.5 | 14.2 | 4.7 | 4.2 | 8.9 | - | 7.6 | 2.5 | 3.6 | 2.8 | 130.4 |
| **MG7** | Distance (Å) | 5.8 | 2.9 | 8.1 | 19.9 | 10.5 | 3.4 | 15.3 | 7.3 | - | 2.2 | 1.8 | 0.9 | 28.4 |
| **MG7** | Angle (°) | 4.1 | 3.6 | 11.6 | 16.2 | 7.6 | 4.9 | 12.9 | 8.6 | - | 2.9 | 2.0 | 1.2 | 43.2 |
| **CUB** | Distance (Å) | 3.3 | 2.6 | 18.3 | 29.6 | 14.7 | 4.7 | 23.8 | 6.5 | 0.9 | - | 0.9 | 1.3 | 58.7 |
| **CUB** | Angle (°) | 2.2 | 2.6 | 16.1 | 18.6 | 9.0 | 4.7 | 17.2 | 6.2 | 0.9 | - | 1.1 | 2.2 | 71.6 |
| **C5d** | Distance (Å) | 3.3 | 2.3 | 19.0 | 23.1 | 11.1 | 1.6 | 18.3 | 3.7 | 2.8 | 1.6 | - | 0.5 | 91.6 |
| **C5d** | Angle (°) | 2.3 | 3.6 | 20.3 | 17.3 | 8.1 | 1.8 | 16.6 | 4.9 | 3.1 | 2.8 | - | 0.9 | 92.6 |
| **MG8** | Distance (Å) | 3.4 | 3.9 | 9.3 | 22.2 | 11.0 | 4.7 | 16.0 | 4.4 | 2.1 | 0.7 | 0.6 | - | 65.2 |
| **MG8** | Angle (°) | 2.0 | 4.8 | 13.1 | 17.1 | 7.6 | 5.4 | 13.2 | 8.5 | 3.4 | 1.1 | 1.1 | - | 104.5 |
| **C345C** | Distance (Å) | 37.9 | 42.0 | 40.1 | 42.2 | 37.2 | 44.4 | 36.9 | 47.6 | 41.1 | 40.5 | 42.1 | 42.0 | - |
| **C345C** | Angle (°) | 19.8 | 34.4 | 28.5 | 21.0 | 18.2 | 39.3 | 20.1 | 44.3 | 73.8 | 43.3 | 38.1 | 61.2 | - |
| **Domain superimposition RMSD (Å)** | | 1.1 | 0.5 | 1.0 | 0.7 | 1.1 | 1.9 | 2.8 | 2.8 | 1.3 | 0.6 | 1.1 | 0.8 | 2.9 |

**Supplementary Table 2. Domain displacement in C5-CVF-SSL7 complex relative to apo C5**. Centre-of-mass translational (Å) and rotational (°) displacement when each C5-CVF-SSL7 domain is superimposed onto each apo C5 domain. The Cα root-mean-square deviation (RMSD) for each domain superimposition is shown below.

| **Complex** | **Metric** | **Apo C5** | | | | | | | | | | | | |
| --- | --- | --- | --- | --- | --- | --- | --- | --- | --- | --- | --- | --- | --- | --- |
|  |  | **MG1** | **MG2** | **MG3** | **MG4** | **MG5** | **MG6** | **Linker** | **C5a** | **MG7** | **CUB** | **C5d** | **MG8** | **C345C** |
| **MG1** | Distance (Å) | - | 1.0 | 23.6 | 4.2 | 1.2 | 5.0 | 0.9 | 13.2 | 17.0 | 8.3 | 11.0 | 8.7 | 112.6 |
| **MG1** | Angle (°) | - | 1.5 | 21.8 | 5.8 | 2.7 | 6.3 | 2.6 | 10.1 | 12.2 | 5.6 | 9.4 | 6.2 | 65.1 |
| **MG2** | Distance (Å) | 2.3 | - | 14.1 | 9.6 | 1.3 | 0.9 | 4.2 | 6.1 | 7.9 | 4.1 | 4.1 | 3.2 | 76.3 |
| **MG2** | Angle (°) | 3.6 | - | 22.5 | 12.3 | 1.7 | 2.6 | 7.8 | 7.8 | 10.0 | 3.9 | 6.6 | 4.1 | 66.7 |
| **MG3** | Distance (Å) | 6.2 | 2.9 | - | 6.1 | 4.3 | 3.3 | 4.6 | 3.4 | 6.4 | 3.3 | 3.2 | 2.9 | 102.5 |
| **MG3** | Angle (°) | 5.6 | 2.8 | - | 8.8 | 4.4 | 4.9 | 6.1 | 7.3 | 9.2 | 2.7 | 2.9 | 2.0 | 117.1 |
| **MG4** | Distance (Å) | 1.5 | 3.0 | 8.8 | - | 0.8 | 5.7 | 2.1 | 15.4 | 17.0 | 8.5 | 11.9 | 10.2 | 147.7 |
| **MG4** | Angle (°) | 2.0 | 3.9 | 12.8 | - | 1.1 | 7.0 | 5.2 | 15.0 | 13.6 | 5.0 | 8.9 | 7.9 | 103.7 |
| **MG5** | Distance (Å) | 0.9 | 2.9 | 18.9 | 3.3 | - | 4.6 | 2.4 | 18.2 | 20.1 | 10.7 | 14.4 | 12.1 | 128.6 |
| **MG5** | Angle (°) | 2.0 | 3.8 | 19.5 | 7.4 | - | 5.8 | 7.4 | 14.0 | 14.9 | 6.3 | 10.6 | 8.3 | 77.3 |
| **MG6** | Distance (Å) | 4.0 | 0.1 | 13.0 | 10.2 | 2.8 | - | 6.2 | 9.8 | 7.9 | 4.0 | 5.2 | 4.2 | 59.7 |
| **MG6** | Angle (°) | 5.0 | 0.2 | 20.9 | 13.0 | 3.3 | - | 9.6 | 10.8 | 12.3 | 1.8 | 5.8 | 4.7 | 56.2 |
| **Linker** | Distance (Å) | 0.9 | 1.9 | 15.8 | 3.2 | 1.1 | 3.7 | - | 13.2 | 15.7 | 7.9 | 10.5 | 8.7 | 120.3 |
| **Linker** | Angle (°) | 2.5 | 3.1 | 20.7 | 8.2 | 1.7 | 5.2 | - | 12.8 | 13.1 | 5.2 | 9.6 | 7.2 | 81.1 |
| **C5a** | Distance (Å) | 3.8 | 3.2 | 9.7 | 13.3 | 3.3 | 4.5 | 7.2 | - | 6.3 | 2.9 | 3.0 | 2.3 | 107.9 |
| **C5a** | Angle (°) | 2.4 | 3.8 | 22.2 | 13.0 | 1.9 | 5.0 | 6.9 | - | 7.4 | 2.7 | 4.0 | 3.6 | 130.2 |
| **MG7** | Distance (Å) | 7.3 | 4.3 | 8.3 | 18.3 | 7.1 | 4.1 | 13.8 | 6.4 | - | 2.6 | 1.4 | 1.1 | 29.1 |
| **MG7** | Angle (°) | 5.0 | 5.3 | 12.0 | 14.8 | 4.5 | 5.1 | 11.5 | 7.6 | - | 3.5 | 1.4 | 1.3 | 44.9 |
| **CUB** | Distance (Å) | 5.1 | 5.3 | 19.0 | 27.2 | 8.1 | 7.8 | 18.0 | 5.3 | 0.8 | - | 1.0 | 1.1 | 57.7 |
| **CUB** | Angle (°) | 3.2 | 5.5 | 16.9 | 17.1 | 4.5 | 7.6 | 13.0 | 5.1 | 1.0 | - | 0.9 | 1.8 | 69.7 |
| **C5d** | Distance (Å) | 3.0 | 2.9 | 19.1 | 20.4 | 5.3 | 1.8 | 12.6 | 2.7 | 2.7 | 2.0 | - | 0.2 | 91.3 |
| **C5d** | Angle (°) | 2.2 | 4.6 | 20.6 | 15.3 | 3.9 | 1.6 | 11.5 | 3.5 | 3.0 | 3.5 | - | 0.4 | 92.0 |
| **MG8** | Distance (Å) | 2.9 | 4.6 | 9.4 | 20.4 | 5.8 | 5.5 | 13.2 | 3.9 | 2.0 | 0.7 | 0.4 | - | 65.0 |
| **MG8** | Angle (°) | 1.8 | 6.0 | 13.4 | 15.8 | 4.0 | 6.3 | 11.0 | 7.6 | 3.0 | 1.2 | 0.7 | - | 104.1 |
| **C345C** | Distance (Å) | 35.2 | 40.3 | 39.0 | 42.1 | 36.1 | 45.6 | 35.6 | 47.3 | 40.7 | 39.7 | 41.5 | 41.3 | - |
| **C345C** | Angle (°) | 18.5 | 33.1 | 27.5 | 21.4 | 17.7 | 41.3 | 20.0 | 44.2 | 72.8 | 42.1 | 37.4 | 60.2 | - |
| **Domain superimposition RMSD (Å)** | | 1.1 | 0.5 | 1.0 | 0.7 | 1.1 | 1.6 | 2.7 | 2.8 | 1.3 | 0.6 | 1.0 | 0.7 | 2.9 |

**Supplementary Table 3. Domain displacement in C5-CVF-H1H complex relative to apo C5**. Centre-of-mass translational (Å) and rotational (°) displacement when each C5-CVF-H1H domain is superimposed onto each apo C5 domain. The data for the C345C domain is absent as it could not be resolved in the crystal structure. The Cα root-mean-square deviation (RMSD) for each domain superimposition is shown below.

| **Complex** | **Metric** | **Apo C5** | | | | | | | | | | | |
| --- | --- | --- | --- | --- | --- | --- | --- | --- | --- | --- | --- | --- | --- |
|  |  | **MG1** | **MG2** | **MG3** | **MG4** | **MG5** | **MG6** | **Linker** | **C5a** | **MG7** | **CUB** | **C5d** | **MG8** |
| **MG1** | Distance (Å) | - | 1.4 | 25.6 | 4.0 | 0.7 | 3.1 | 1.1 | 23.8 | 18.2 | 8.8 | 11.9 | 10.2 |
| **MG1** | Angle (°) | - | 1.8 | 23.5 | 5.5 | 1.3 | 3.8 | 3.2 | 18.3 | 13.2 | 6.0 | 10.1 | 7.1 |
| **MG2** | Distance (Å) | 1.5 | - | 14.3 | 9.8 | 1.4 | 0.1 | 1.2 | 11.0 | 7.4 | 2.7 | 3.6 | 2.8 |
| **MG2** | Angle (°) | 1.9 | - | 22.8 | 12.7 | 1.9 | 0.3 | 2.2 | 14.1 | 9.4 | 2.1 | 5.8 | 3.6 |
| **MG3** | Distance (Å) | 4.4 | 2.4 | - | 8.1 | 3.2 | 2.7 | 2.8 | 5.4 | 6.9 | 3.0 | 3.2 | 3.1 |
| **MG3** | Angle (°) | 3.7 | 1.7 | - | 11.7 | 3.1 | 3.4 | 3.4 | 12.3 | 9.8 | 1.8 | 2.2 | 0.9 |
| **MG4** | Distance (Å) | 1.9 | 1.5 | 9.1 | - | 1.5 | 3.6 | 2.0 | 20.7 | 17.2 | 7.4 | 10.4 | 9.1 |
| **MG4** | Angle (°) | 2.6 | 1.7 | 13.3 | - | 2.5 | 4.4 | 4.7 | 20.3 | 14.0 | 4.3 | 7.8 | 7.0 |
| **MG5** | Distance (Å) | 0.6 | 2.3 | 20.6 | 3.4 | - | 2.5 | 0.8 | 27.6 | 21.1 | 10.7 | 14.2 | 12.5 |
| **MG5** | Angle (°) | 0.9 | 3.0 | 21.1 | 7.3 | - | 3.2 | 2.0 | 21.2 | 15.8 | 6.4 | 10.4 | 8.6 |
| **MG6** | Distance (Å) | 2.4 | 0.1 | 13.4 | 11.0 | 1.9 | - | 2.4 | 15.9 | 8.1 | 4.0 | 5.2 | 4.2 |
| **MG6** | Angle (°) | 3.0 | 0.4 | 21.1 | 13.9 | 2.3 | - | 3.3 | 17.5 | 12.9 | 1.3 | 5.9 | 4.6 |
| **Linker** | Distance (Å) | 0.6 | 1.5 | 17.8 | 3.0 | 1.0 | 2.5 | - | 21.5 | 16.9 | 7.8 | 10.9 | 9.4 |
| **Linker** | Angle (°) | 1.6 | 2.6 | 22.1 | 7.4 | 3.3 | 3.4 | - | 20.1 | 13.8 | 5.2 | 9.6 | 7.5 |
| **C5a** | Distance (Å) | 3.2 | 3.7 | 9.8 | 15.6 | 6.1 | 6.2 | 4.3 | - | 6.6 | 2.5 | 2.7 | 2.5 |
| **C5a** | Angle (°) | 0.9 | 4.6 | 22.7 | 15.1 | 4.2 | 6.8 | 3.4 | - | 7.9 | 2.1 | 3.5 | 3.4 |
| **MG7** | Distance (Å) | 6.5 | 4.9 | 7.7 | 21.2 | 8.3 | 5.5 | 8.1 | 11.7 | - | 1.9 | 1.5 | 0.9 |
| **MG7** | Angle (°) | 4.6 | 6.3 | 10.9 | 17.4 | 5.8 | 7.9 | 6.4 | 14.0 | - | 2.4 | 1.5 | 1.4 |
| **CUB** | Distance (Å) | 7.7 | 7.0 | 19.0 | 28.7 | 11.2 | 10.0 | 11.6 | 7.8 | 1.6 | - | 0.3 | 0.9 |
| **CUB** | Angle (°) | 5.2 | 7.8 | 16.8 | 18.0 | 6.7 | 10.2 | 8.1 | 7.5 | 2.1 | - | 0.6 | 1.5 |
| **C5d** | Distance (Å) | 4.5 | 2.8 | 18.9 | 20.6 | 6.9 | 4.2 | 6.7 | 3.2 | 1.2 | 1.7 | - | 0.6 |
| **C5d** | Angle (°) | 3.4 | 4.4 | 20.2 | 15.5 | 5.0 | 4.8 | 5.8 | 4.2 | 1.1 | 3.3 | - | 0.9 |
| **MG8** | Distance (Å) | 5.5 | 5.7 | 10.1 | 23.0 | 9.3 | 8.0 | 8.2 | 5.6 | 1.7 | 1.0 | 0.3 | - |
| **MG8** | Angle (°) | 3.7 | 7.5 | 14.2 | 17.8 | 6.4 | 9.2 | 6.5 | 11.3 | 2.7 | 1.5 | 0.0 | - |
| **Domain superimposition RMSD (Å)** | | 1.7 | 0.6 | 1.6 | 1.0 | 1.3 | 1.0 | 2.2 | 5.1 | 1.0 | 1.2 | 1.3 | 0.6 |

**Supplementary Table 4. Domain displacement in C5-OmCI-RaCI1 complex relative to apo C5**. Centre-of-mass translational (Å) and rotational (°) displacement when each C5-OmCI-RaCI1 domain is superimposed onto each apo C5 domain. The Cα root-mean-square deviation (RMSD) for each domain superimposition is shown below.

| **Complex** | **Metric** | **Apo C5** | | | | | | | | | | | | |
| --- | --- | --- | --- | --- | --- | --- | --- | --- | --- | --- | --- | --- | --- | --- |
|  |  | **MG1** | **MG2** | **MG3** | **MG4** | **MG5** | **MG6** | **Linker** | **C5a** | **MG7** | **CUB** | **C5d** | **MG8** | **C345C** |
| **MG1** | Distance (Å) | - | 0.8 | 11.5 | 2.3 | 0.9 | 2.0 | 2.2 | 15.7 | 6.4 | 4.6 | 3.4 | 2.2 | 113.0 |
| **MG1** | Angle (°) | - | 0.7 | 10.7 | 3.1 | 2.0 | 2.4 | 6.5 | 12.3 | 4.5 | 3.1 | 2.8 | 1.4 | 64.7 |
| **MG2** | Distance (Å) | 1.3 | - | 8.4 | 3.2 | 1.7 | 0.2 | 2.8 | 9.5 | 3.4 | 2.9 | 1.5 | 0.6 | 75.0 |
| **MG2** | Angle (°) | 1.8 | - | 13.1 | 3.7 | 2.1 | 0.6 | 4.7 | 12.5 | 4.2 | 3.1 | 2.4 | 0.7 | 65.0 |
| **MG3** | Distance (Å) | 3.7 | 4.5 | - | 0.9 | 2.5 | 3.8 | 8.6 | 6.4 | 5.4 | 4.1 | 4.9 | 4.8 | 102.1 |
| **MG3** | Angle (°) | 3.4 | 6.5 | - | 1.4 | 2.6 | 6.1 | 10.6 | 12.8 | 7.6 | 2.7 | 3.3 | 4.0 | 114.8 |
| **MG4** | Distance (Å) | 1.7 | 4.2 | 2.3 | - | 1.1 | 2.9 | 4.7 | 16.1 | 9.0 | 6.1 | 7.1 | 5.5 | 149.8 |
| **MG4** | Angle (°) | 2.2 | 5.1 | 3.3 | - | 2.4 | 3.6 | 11.3 | 15.5 | 7.2 | 3.7 | 5.0 | 3.6 | 102.8 |
| **MG5** | Distance (Å) | 0.7 | 2.4 | 7.5 | 1.1 | - | 1.0 | 2.4 | 18.8 | 8.6 | 6.0 | 6.0 | 3.4 | 130.5 |
| **MG5** | Angle (°) | 1.5 | 3.0 | 7.7 | 2.4 | - | 1.3 | 8.3 | 14.7 | 6.3 | 3.7 | 4.4 | 2.2 | 77.2 |
| **MG6** | Distance (Å) | 2.6 | 0.2 | 7.3 | 1.9 | 0.8 | - | 2.3 | 12.5 | 3.0 | 2.8 | 1.8 | 0.3 | 59.6 |
| **MG6** | Angle (°) | 3.2 | 0.4 | 11.7 | 2.4 | 1.1 | - | 3.3 | 13.8 | 4.7 | 2.6 | 2.0 | 0.2 | 54.5 |
| **Linker** | Distance (Å) | 1.0 | 1.2 | 6.6 | 0.6 | 0.6 | 1.2 | - | 14.2 | 6.5 | 4.6 | 4.0 | 2.5 | 125.6 |
| **Linker** | Angle (°) | 2.7 | 1.6 | 8.2 | 1.1 | 0.9 | 1.7 | - | 13.5 | 5.2 | 3.2 | 3.3 | 1.6 | 80.9 |
| **C5a** | Distance (Å) | 3.5 | 1.4 | 8.9 | 5.9 | 3.7 | 1.3 | 7.0 | - | 3.9 | 2.8 | 2.7 | 1.9 | 107.0 |
| **C5a** | Angle (°) | 2.7 | 1.8 | 18.8 | 5.3 | 2.8 | 1.4 | 6.6 | - | 4.6 | 2.5 | 3.4 | 2.2 | 129.7 |
| **MG7** | Distance (Å) | 3.4 | 0.6 | 4.7 | 2.7 | 3.1 | 1.8 | 6.9 | 11.5 | - | 1.7 | 0.6 | 0.7 | 27.9 |
| **MG7** | Angle (°) | 2.4 | 0.7 | 6.5 | 2.1 | 2.3 | 2.9 | 5.5 | 13.6 | - | 2.3 | 0.6 | 1.1 | 43.2 |
| **CUB** | Distance (Å) | 1.1 | 1.5 | 14.1 | 10.0 | 6.9 | 3.6 | 7.4 | 8.9 | 0.9 | - | 0.5 | 2.2 | 56.9 |
| **CUB** | Angle (°) | 0.7 | 1.4 | 12.2 | 6.2 | 4.2 | 3.7 | 5.2 | 8.5 | 1.1 | - | 0.4 | 3.6 | 69.3 |
| **C5d** | Distance (Å) | 1.1 | 0.6 | 14.6 | 8.6 | 5.1 | 1.7 | 7.3 | 4.6 | 1.6 | 1.8 | - | 1.2 | 89.5 |
| **C5d** | Angle (°) | 0.5 | 0.9 | 14.9 | 6.1 | 3.7 | 1.9 | 6.4 | 6.0 | 1.7 | 3.5 | - | 2.1 | 90.0 |
| **MG8** | Distance (Å) | 2.0 | 1.1 | 7.6 | 6.9 | 5.5 | 2.9 | 8.4 | 6.0 | 0.7 | 0.5 | 0.3 | - | 62.1 |
| **MG8** | Angle (°) | 1.2 | 1.3 | 9.4 | 4.9 | 3.6 | 3.3 | 6.6 | 12.1 | 1.1 | 0.7 | 0.5 | - | 102.3 |
| **C345C** | Distance (Å) | 39.8 | 40.7 | 40.6 | 42.9 | 43.2 | 42.5 | 35.4 | 47.5 | 39.7 | 39.2 | 40.8 | 41.9 | - |
| **C345C** | Angle (°) | 20.8 | 33.1 | 29.1 | 20.1 | 21.2 | 36.8 | 18.1 | 44.6 | 71.9 | 41.9 | 37.0 | 63.4 | - |
| **Domain superimposition RMSD (Å)** | | 1.7 | 0.5 | 1.3 | 0.6 | 1.4 | 0.7 | 2.9 | 4.9 | 1.0 | 1.0 | 1.2 | 1.7 | 3.1 |

**Supplementary Table 5. Domain displacement in C5-OmCI-RaCI2 complex relative to apo C5**. Centre-of-mass translational (Å) and rotational (°) displacement when each C5-OmCI-RaCI2 domain is superimposed onto each apo C5 domain. The Cα root-mean-square deviation (RMSD) for each domain superimposition is shown below.

| **Complex** | **Metric** | **Apo C5** | | | | | | | | | | | | |
| --- | --- | --- | --- | --- | --- | --- | --- | --- | --- | --- | --- | --- | --- | --- |
|  |  | **MG1** | **MG2** | **MG3** | **MG4** | **MG5** | **MG6** | **Linker** | **C5a** | **MG7** | **CUB** | **C5d** | **MG8** | **C345C** |
| **MG1** | Distance (Å) | - | 0.8 | 11.3 | 2.3 | 0.9 | 1.8 | 2.2 | 14.0 | 5.8 | 4.3 | 2.6 | 2.3 | 112.9 |
| **MG1** | Angle (°) | - | 0.7 | 10.5 | 3.2 | 2.1 | 2.2 | 6.6 | 11.1 | 4.1 | 3.0 | 2.1 | 1.5 | 64.7 |
| **MG2** | Distance (Å) | 1.3 | - | 8.3 | 3.3 | 1.6 | 0.3 | 2.9 | 8.8 | 3.1 | 2.5 | 1.2 | 0.9 | 74.8 |
| **MG2** | Angle (°) | 1.7 | - | 13.0 | 3.8 | 2.0 | 0.8 | 4.9 | 11.6 | 3.9 | 2.7 | 1.8 | 1.1 | 64.9 |
| **MG3** | Distance (Å) | 3.6 | 4.3 | - | 0.9 | 2.5 | 3.6 | 8.6 | 6.1 | 5.3 | 4.2 | 4.8 | 4.7 | 102.0 |
| **MG3** | Angle (°) | 3.3 | 6.2 | - | 1.3 | 2.6 | 5.8 | 10.6 | 12.2 | 7.3 | 2.7 | 3.0 | 4.0 | 114.7 |
| **MG4** | Distance (Å) | 1.7 | 4.2 | 2.2 | - | 1.2 | 2.7 | 4.8 | 15.0 | 8.4 | 5.9 | 6.4 | 4.9 | 149.9 |
| **MG4** | Angle (°) | 2.4 | 5.1 | 3.1 | - | 2.7 | 3.4 | 11.5 | 14.5 | 6.7 | 3.6 | 4.4 | 3.2 | 102.8 |
| **MG5** | Distance (Å) | 0.8 | 2.4 | 7.2 | 1.1 | - | 0.7 | 2.4 | 17.3 | 8.0 | 5.7 | 5.1 | 2.7 | 130.6 |
| **MG5** | Angle (°) | 1.7 | 3.1 | 7.5 | 2.5 | - | 0.9 | 8.3 | 13.5 | 5.8 | 3.5 | 3.7 | 1.7 | 77.2 |
| **MG6** | Distance (Å) | 2.5 | 0.3 | 7.2 | 1.9 | 0.8 | - | 2.3 | 11.7 | 2.7 | 2.5 | 1.3 | 0.3 | 59.7 |
| **MG6** | Angle (°) | 3.0 | 0.5 | 11.6 | 2.4 | 1.0 | - | 3.3 | 12.9 | 4.2 | 2.4 | 1.5 | 0.3 | 54.5 |
| **Linker** | Distance (Å) | 0.9 | 1.3 | 6.5 | 0.6 | 0.6 | 1.0 | - | 12.9 | 6.0 | 4.4 | 3.2 | 2.2 | 125.6 |
| **Linker** | Angle (°) | 2.5 | 1.6 | 8.0 | 1.2 | 1.0 | 1.3 | - | 12.3 | 4.7 | 3.1 | 2.7 | 1.4 | 80.9 |
| **C5a** | Distance (Å) | 3.5 | 1.5 | 8.9 | 6.1 | 3.5 | 1.1 | 6.8 | - | 3.9 | 2.8 | 2.6 | 1.8 | 106.9 |
| **C5a** | Angle (°) | 2.7 | 1.8 | 19.0 | 5.6 | 2.7 | 1.1 | 6.4 | - | 4.6 | 2.5 | 3.3 | 2.0 | 129.5 |
| **MG7** | Distance (Å) | 3.3 | 0.3 | 4.6 | 2.5 | 2.9 | 1.7 | 6.5 | 10.9 | - | 1.6 | 0.5 | 0.6 | 27.8 |
| **MG7** | Angle (°) | 2.4 | 0.3 | 6.3 | 1.9 | 2.1 | 2.7 | 5.2 | 13.0 | - | 2.1 | 0.4 | 1.0 | 43.1 |
| **CUB** | Distance (Å) | 0.7 | 1.3 | 13.7 | 9.7 | 6.4 | 3.4 | 7.0 | 8.8 | 0.9 | - | 0.5 | 2.2 | 56.9 |
| **CUB** | Angle (°) | 0.4 | 1.1 | 11.8 | 6.0 | 3.9 | 3.4 | 4.9 | 8.5 | 1.1 | - | 0.6 | 3.5 | 69.4 |
| **C5d** | Distance (Å) | 1.1 | 0.2 | 14.4 | 8.7 | 4.8 | 1.7 | 7.0 | 4.6 | 1.6 | 1.7 | - | 1.2 | 89.3 |
| **C5d** | Angle (°) | 0.5 | 0.2 | 14.7 | 6.2 | 3.5 | 1.9 | 6.1 | 6.0 | 1.7 | 3.1 | - | 2.1 | 89.8 |
| **MG8** | Distance (Å) | 2.1 | 0.4 | 7.5 | 6.9 | 5.1 | 2.7 | 8.0 | 5.9 | 0.7 | 0.4 | 0.3 | - | 62.1 |
| **MG8** | Angle (°) | 1.4 | 0.5 | 9.3 | 4.8 | 3.4 | 3.1 | 6.3 | 11.8 | 1.1 | 0.6 | 0.5 | - | 102.1 |
| **C345C** | Distance (Å) | 40.3 | 41.1 | 40.8 | 43.2 | 43.5 | 42.6 | 35.4 | 47.5 | 39.7 | 39.2 | 40.8 | 41.9 | - |
| **C345C** | Angle (°) | 21.0 | 33.6 | 29.3 | 20.3 | 21.3 | 36.7 | 18.1 | 44.6 | 72.1 | 41.9 | 37.0 | 63.3 | - |
| **Domain superimposition RMSD (Å)** | | 1.7 | 0.5 | 1.3 | 0.6 | 1.4 | 0.7 | 2.9 | 4.9 | 1.0 | 1.0 | 1.2 | 1.7 | 3.1 |

**Supplementary Table 6. Domain displacement in C5-OmCI-RaCI3 complex relative to apo C5**. Centre-of-mass translational (Å) and rotational (°) displacement when each C5-OmCI-RaCI3 domain is superimposed onto each apo C5 domain. The Cα root-mean-square deviation (RMSD) for each domain superimposition is shown below.

| **Complex** | **Metric** | **Apo C5** | | | | | | | | | | | | |
| --- | --- | --- | --- | --- | --- | --- | --- | --- | --- | --- | --- | --- | --- | --- |
|  |  | **MG1** | **MG2** | **MG3** | **MG4** | **MG5** | **MG6** | **Linker** | **C5a** | **MG7** | **CUB** | **C5d** | **MG8** | **C345C** |
| **MG1** | Distance (Å) | - | 0.8 | 11.5 | 2.1 | 0.9 | 2.1 | 2.3 | 15.6 | 6.4 | 4.9 | 3.2 | 2.2 | 113.0 |
| **MG1** | Angle (°) | - | 0.4 | 10.6 | 2.8 | 2.2 | 2.5 | 6.8 | 12.3 | 4.6 | 3.4 | 2.6 | 1.2 | 64.6 |
| **MG2** | Distance (Å) | 1.4 | - | 8.4 | 3.3 | 1.9 | 0.2 | 2.8 | 9.5 | 3.5 | 3.2 | 1.5 | 0.6 | 74.9 |
| **MG2** | Angle (°) | 1.8 | - | 13.1 | 3.7 | 2.2 | 0.6 | 4.6 | 12.4 | 4.4 | 3.5 | 2.4 | 0.2 | 64.8 |
| **MG3** | Distance (Å) | 3.6 | 4.7 | - | 1.1 | 2.5 | 3.9 | 8.5 | 6.4 | 5.5 | 4.4 | 4.8 | 4.8 | 102.0 |
| **MG3** | Angle (°) | 3.2 | 6.8 | - | 1.5 | 2.6 | 6.3 | 10.5 | 12.6 | 7.7 | 3.0 | 3.3 | 3.9 | 114.6 |
| **MG4** | Distance (Å) | 1.6 | 4.4 | 2.3 | - | 1.0 | 3.1 | 4.6 | 16.0 | 9.1 | 6.6 | 7.0 | 5.7 | 149.8 |
| **MG4** | Angle (°) | 2.1 | 5.2 | 3.3 | - | 2.3 | 3.9 | 11.1 | 15.4 | 7.2 | 4.0 | 4.9 | 3.7 | 102.6 |
| **MG5** | Distance (Å) | 0.7 | 2.6 | 7.6 | 1.1 | - | 1.1 | 2.3 | 18.8 | 8.7 | 6.4 | 5.9 | 3.7 | 130.5 |
| **MG5** | Angle (°) | 1.7 | 3.3 | 7.8 | 2.4 | - | 1.5 | 8.2 | 14.7 | 6.4 | 3.9 | 4.3 | 2.2 | 77.0 |
| **MG6** | Distance (Å) | 2.6 | 0.1 | 7.3 | 1.9 | 0.8 | - | 2.3 | 12.4 | 2.9 | 3.0 | 1.7 | 0.5 | 59.5 |
| **MG6** | Angle (°) | 3.2 | 0.1 | 11.7 | 2.4 | 1.1 | - | 3.2 | 13.7 | 4.7 | 2.9 | 1.9 | 0.5 | 54.3 |
| **Linker** | Distance (Å) | 0.9 | 1.5 | 6.7 | 0.6 | 0.6 | 1.3 | - | 14.2 | 6.6 | 5.0 | 3.9 | 2.6 | 125.6 |
| **Linker** | Angle (°) | 2.5 | 1.9 | 8.2 | 1.1 | 0.9 | 1.8 | - | 13.5 | 5.3 | 3.5 | 3.3 | 1.5 | 80.7 |
| **C5a** | Distance (Å) | 3.5 | 1.7 | 8.8 | 5.8 | 3.8 | 1.3 | 7.0 | - | 4.0 | 3.0 | 2.6 | 2.0 | 107.0 |
| **C5a** | Angle (°) | 2.7 | 2.2 | 18.5 | 5.1 | 2.9 | 1.4 | 6.6 | - | 4.7 | 2.7 | 3.3 | 2.3 | 129.7 |
| **MG7** | Distance (Å) | 3.4 | 0.8 | 4.7 | 2.7 | 3.1 | 1.7 | 6.9 | 11.3 | - | 1.9 | 0.6 | 1.0 | 27.8 |
| **MG7** | Angle (°) | 2.4 | 1.0 | 6.5 | 2.1 | 2.3 | 2.7 | 5.5 | 13.4 | - | 2.5 | 0.4 | 1.5 | 42.9 |
| **CUB** | Distance (Å) | 1.2 | 1.5 | 14.1 | 9.9 | 7.0 | 3.4 | 7.7 | 8.8 | 1.1 | - | 0.5 | 2.1 | 56.8 |
| **CUB** | Angle (°) | 0.8 | 1.3 | 12.2 | 6.1 | 4.3 | 3.4 | 5.4 | 8.4 | 1.3 | - | 0.5 | 3.4 | 69.2 |
| **C5d** | Distance (Å) | 1.3 | 1.1 | 14.6 | 8.6 | 5.3 | 1.6 | 7.6 | 4.5 | 1.8 | 1.7 | - | 1.1 | 89.3 |
| **C5d** | Angle (°) | 0.5 | 1.7 | 15.0 | 6.1 | 3.8 | 1.8 | 6.7 | 5.9 | 1.9 | 3.2 | - | 2.0 | 89.8 |
| **MG8** | Distance (Å) | 2.2 | 1.3 | 7.5 | 6.9 | 5.6 | 2.8 | 8.6 | 6.0 | 0.7 | 0.5 | 0.3 | - | 62.1 |
| **MG8** | Angle (°) | 1.2 | 1.6 | 9.2 | 4.7 | 3.6 | 3.2 | 6.6 | 12.0 | 1.0 | 0.8 | 0.5 | - | 102.3 |
| **C345C** | Distance (Å) | 40.0 | 39.9 | 40.6 | 42.7 | 43.1 | 42.3 | 35.3 | 47.3 | 39.7 | 39.1 | 40.8 | 41.9 | - |
| **C345C** | Angle (°) | 20.8 | 32.4 | 29.0 | 19.8 | 21.0 | 36.5 | 17.9 | 44.4 | 72.1 | 41.7 | 37.0 | 63.4 | - |
| **Domain superimposition RMSD (Å)** | | 1.6 | 0.5 | 1.3 | 0.6 | 1.4 | 0.7 | 3.0 | 4.9 | 1.0 | 1.0 | 1.2 | 1.7 | 3.1 |

**Supplementary Table 7. Domain displacement in C5-OmCI-RaCI1-CirpT1 complex relative to apo C5**. Centre-of-mass translational (Å) and rotational (°) displacement when each C5-OmCI-RaCI1-CirpT1 domain is superimposed onto each apo C5 domain. The Cα root-mean-square deviation (RMSD) for each domain superimposition is shown below.

| **Complex** | **Metric** | **Apo C5** | | | | | | | | | | | | |
| --- | --- | --- | --- | --- | --- | --- | --- | --- | --- | --- | --- | --- | --- | --- |
|  |  | **MG1** | **MG2** | **MG3** | **MG4** | **MG5** | **MG6** | **Linker** | **C5a** | **MG7** | **CUB** | **C5d** | **MG8** | **C345C** |
| **MG1** | Distance (Å) | - | 0.4 | 14.8 | 3.2 | 1.0 | 0.7 | 1.0 | 13.6 | 5.0 | 3.1 | 3.6 | 2.0 | 118.2 |
| **MG1** | Angle (°) | - | 0.2 | 13.8 | 4.4 | 2.3 | 0.8 | 3.0 | 10.7 | 3.6 | 2.1 | 3.1 | 1.4 | 68.5 |
| **MG2** | Distance (Å) | 1.0 | - | 9.8 | 2.6 | 1.2 | 0.9 | 1.4 | 8.5 | 2.4 | 1.9 | 1.5 | 1.1 | 78.4 |
| **MG2** | Angle (°) | 1.5 | - | 15.7 | 3.2 | 1.6 | 2.6 | 2.4 | 11.1 | 3.0 | 2.0 | 2.3 | 1.5 | 68.8 |
| **MG3** | Distance (Å) | 3.6 | 4.3 | - | 0.8 | 2.5 | 2.8 | 6.3 | 5.8 | 4.2 | 3.0 | 4.2 | 3.9 | 102.4 |
| **MG3** | Angle (°) | 3.3 | 6.5 | - | 0.6 | 2.6 | 4.3 | 7.7 | 12.1 | 5.9 | 1.9 | 3.0 | 3.7 | 117.9 |
| **MG4** | Distance (Å) | 1.4 | 3.5 | 2.8 | - | 1.1 | 1.1 | 3.3 | 13.4 | 6.4 | 3.9 | 5.9 | 3.4 | 152.2 |
| **MG4** | Angle (°) | 1.9 | 4.4 | 3.9 | - | 2.6 | 0.9 | 7.9 | 13.2 | 5.2 | 2.4 | 4.3 | 2.4 | 106.4 |
| **MG5** | Distance (Å) | 0.5 | 2.0 | 9.6 | 1.6 | - | 0.8 | 1.8 | 16.0 | 6.6 | 4.1 | 5.5 | 1.6 | 134.6 |
| **MG5** | Angle (°) | 0.6 | 2.7 | 10.0 | 3.6 | - | 1.0 | 5.7 | 12.6 | 4.8 | 2.4 | 4.0 | 1.1 | 80.5 |
| **MG6** | Distance (Å) | 2.6 | 0.2 | 9.2 | 1.4 | 0.6 | - | 1.8 | 11.2 | 2.0 | 1.8 | 1.9 | 1.1 | 62.2 |
| **MG6** | Angle (°) | 3.2 | 0.4 | 15.1 | 1.6 | 0.6 | - | 2.5 | 12.3 | 3.1 | 1.4 | 2.1 | 0.8 | 57.3 |
| **Linker** | Distance (Å) | 0.8 | 0.8 | 8.9 | 0.9 | 0.8 | 1.3 | - | 12.5 | 4.8 | 3.1 | 3.7 | 1.5 | 128.9 |
| **Linker** | Angle (°) | 2.6 | 1.3 | 10.9 | 2.1 | 1.2 | 1.7 | - | 11.9 | 3.8 | 2.0 | 3.3 | 1.2 | 84.2 |
| **C5a** | Distance (Å) | 2.3 | 1.9 | 9.5 | 5.5 | 2.9 | 2.0 | 4.1 | - | 3.2 | 2.5 | 2.6 | 1.7 | 106.9 |
| **C5a** | Angle (°) | 1.7 | 2.3 | 21.0 | 5.4 | 2.2 | 1.6 | 3.7 | - | 3.7 | 2.3 | 3.5 | 1.9 | 132.5 |
| **MG7** | Distance (Å) | 2.9 | 1.2 | 5.9 | 1.6 | 3.1 | 2.2 | 4.5 | 10.4 | - | 1.1 | 1.1 | 1.0 | 28.0 |
| **MG7** | Angle (°) | 2.0 | 1.5 | 8.4 | 1.2 | 2.3 | 3.5 | 3.6 | 12.4 | - | 1.5 | 1.2 | 1.5 | 43.6 |
| **CUB** | Distance (Å) | 2.5 | 1.5 | 14.8 | 8.0 | 5.7 | 4.3 | 4.6 | 8.5 | 1.1 | - | 0.4 | 2.3 | 57.6 |
| **CUB** | Angle (°) | 1.7 | 1.3 | 12.9 | 4.9 | 3.5 | 4.5 | 3.1 | 8.3 | 1.4 | - | 0.6 | 3.7 | 70.6 |
| **C5d** | Distance (Å) | 1.1 | 0.4 | 15.8 | 7.2 | 3.8 | 3.0 | 3.5 | 4.6 | 1.4 | 1.4 | - | 1.5 | 91.4 |
| **C5d** | Angle (°) | 0.9 | 0.6 | 16.5 | 5.3 | 2.8 | 3.4 | 3.2 | 6.1 | 1.5 | 2.6 | - | 2.7 | 93.1 |
| **MG8** | Distance (Å) | 1.0 | 1.4 | 7.2 | 5.6 | 4.5 | 3.6 | 5.0 | 5.6 | 0.6 | 0.3 | 0.3 | - | 62.1 |
| **MG8** | Angle (°) | 0.6 | 1.8 | 9.5 | 4.1 | 3.0 | 4.0 | 4.0 | 11.4 | 0.5 | 0.5 | 0.3 | - | 104.5 |
| **C345C** | Distance (Å) | 38.0 | 40.5 | 39.7 | 42.7 | 42.8 | 41.3 | 36.9 | 46.3 | 39.1 | 39.5 | 40.9 | 41.6 | - |
| **C345C** | Angle (°) | 19.9 | 33.2 | 29.4 | 20.8 | 21.3 | 35.6 | 19.9 | 44.3 | 70.8 | 42.3 | 37.1 | 64.0 | - |
| **Domain superimposition RMSD (Å)** | | 1.8 | 0.5 | 1.6 | 0.7 | 1.3 | 0.8 | 2.3 | 4.9 | 1.1 | 1.1 | 1.2 | 1.8 | 3.0 |

**Supplementary Table 8. Domain displacement in C5-Eculizumab complex relative to apo C5**. Centre-of-mass translational (Å) and rotational (°) displacement when each C5-Eculizumab domain is superimposed onto each apo C5 domain. The Cα root-mean-square deviation (RMSD) for each domain superimposition is shown below.

| **Complex** | **Metric** | **Apo C5** | | | | | | | | | | | | |
| --- | --- | --- | --- | --- | --- | --- | --- | --- | --- | --- | --- | --- | --- | --- |
|  |  | **MG1** | **MG2** | **MG3** | **MG4** | **MG5** | **MG6** | **Linker** | **C5a** | **MG7** | **CUB** | **C5d** | **MG8** | **C345C** |
| **MG1** | Distance (Å) | - | 1.1 | 16.9 | 1.9 | 0.8 | 1.1 | 0.4 | 16.5 | 5.9 | 3.8 | 6.9 | 5.4 | 118.1 |
| **MG1** | Angle (°) | - | 1.2 | 15.6 | 2.6 | 1.9 | 1.1 | 1.0 | 12.8 | 4.2 | 2.6 | 5.6 | 3.7 | 67.8 |
| **MG2** | Distance (Å) | 1.9 | - | 10.5 | 2.5 | 1.6 | 0.7 | 2.4 | 8.1 | 3.4 | 2.3 | 2.2 | 1.6 | 78.5 |
| **MG2** | Angle (°) | 2.7 | - | 16.6 | 2.6 | 1.7 | 1.7 | 4.6 | 10.5 | 4.2 | 2.3 | 3.5 | 1.9 | 68.1 |
| **MG3** | Distance (Å) | 5.1 | 5.0 | - | 1.8 | 3.4 | 4.1 | 7.0 | 6.5 | 4.2 | 3.2 | 4.5 | 4.3 | 103.0 |
| **MG3** | Angle (°) | 4.6 | 7.1 | - | 2.5 | 3.5 | 6.7 | 9.1 | 13.1 | 5.9 | 1.6 | 2.9 | 3.4 | 116.5 |
| **MG4** | Distance (Å) | 2.3 | 3.5 | 3.4 | - | 1.0 | 2.7 | 2.7 | 15.7 | 7.0 | 4.0 | 7.2 | 6.3 | 153.0 |
| **MG4** | Angle (°) | 3.1 | 4.0 | 4.9 | - | 2.1 | 3.4 | 6.5 | 15.2 | 5.7 | 2.4 | 5.0 | 4.5 | 106.1 |
| **MG5** | Distance (Å) | 0.3 | 2.1 | 11.4 | 1.0 | - | 1.4 | 1.5 | 19.1 | 7.2 | 3.9 | 7.9 | 6.6 | 135.4 |
| **MG5** | Angle (°) | 0.6 | 2.6 | 11.8 | 2.2 | - | 1.8 | 4.0 | 14.9 | 5.3 | 2.4 | 5.6 | 4.4 | 80.6 |
| **MG6** | Distance (Å) | 3.7 | 0.9 | 9.8 | 1.1 | 0.5 | - | 3.8 | 11.5 | 2.5 | 1.5 | 2.2 | 1.9 | 63.4 |
| **MG6** | Angle (°) | 4.6 | 2.4 | 16.1 | 1.3 | 0.6 | - | 5.9 | 12.8 | 4.0 | 1.2 | 2.5 | 2.1 | 58.2 |
| **Linker** | Distance (Å) | 1.0 | 1.0 | 9.7 | 0.7 | 1.0 | 0.6 | - | 14.3 | 5.5 | 3.2 | 5.6 | 4.5 | 125.5 |
| **Linker** | Angle (°) | 2.8 | 1.8 | 12.7 | 0.9 | 1.2 | 0.5 | - | 13.9 | 4.5 | 2.2 | 5.0 | 3.6 | 83.6 |
| **C5a** | Distance (Å) | 3.0 | 2.2 | 9.6 | 4.6 | 3.7 | 2.3 | 5.6 | - | 2.3 | 3.0 | 3.0 | 2.0 | 106.9 |
| **C5a** | Angle (°) | 2.1 | 2.9 | 20.6 | 4.0 | 2.8 | 2.4 | 5.4 | - | 2.7 | 2.8 | 4.0 | 3.0 | 130.0 |
| **MG7** | Distance (Å) | 5.0 | 2.7 | 7.2 | 3.5 | 4.0 | 2.6 | 8.8 | 10.7 | - | 2.2 | 2.0 | 1.2 | 29.1 |
| **MG7** | Angle (°) | 3.6 | 3.3 | 10.2 | 2.8 | 3.0 | 4.2 | 7.3 | 12.8 | - | 2.8 | 2.1 | 2.0 | 44.6 |
| **CUB** | Distance (Å) | 3.4 | 2.1 | 15.4 | 8.1 | 5.7 | 3.1 | 9.4 | 6.5 | 1.0 | - | 0.7 | 1.0 | 57.4 |
| **CUB** | Angle (°) | 2.4 | 2.1 | 13.4 | 5.0 | 3.5 | 3.1 | 6.8 | 6.3 | 0.9 | - | 0.6 | 1.7 | 69.0 |
| **C5d** | Distance (Å) | 3.3 | 2.3 | 16.1 | 6.1 | 4.0 | 1.8 | 7.7 | 2.4 | 2.1 | 1.9 | - | 0.2 | 91.4 |
| **C5d** | Angle (°) | 2.1 | 3.6 | 16.7 | 4.1 | 2.7 | 2.0 | 6.9 | 3.1 | 2.2 | 3.5 | - | 0.4 | 92.0 |
| **MG8** | Distance (Å) | 3.1 | 2.9 | 7.9 | 6.3 | 5.6 | 3.5 | 8.8 | 4.9 | 0.4 | 1.1 | 0.8 | - | 63.9 |
| **MG8** | Angle (°) | 1.8 | 3.7 | 10.3 | 4.5 | 3.7 | 4.0 | 7.2 | 9.9 | 0.6 | 1.7 | 1.5 | - | 102.9 |
| **C345C** | Distance (Å) | 37.3 | 39.7 | 40.6 | 43.3 | 42.8 | 42.5 | 36.0 | 49.0 | 40.8 | 40.4 | 41.2 | 42.1 | - |
| **C345C** | Angle (°) | 19.1 | 32.0 | 28.8 | 20.3 | 20.8 | 36.5 | 19.1 | 46.5 | 73.0 | 43.3 | 37.1 | 61.9 | - |
| **Domain superimposition RMSD (Å)** | | 1.8 | 0.5 | 1.3 | 0.7 | 0.9 | 1.0 | 2.3 | 4.2 | 1.2 | 1.0 | 1.1 | 0.9 | 2.3 |

**Supplementary Table 9. Domain displacement in C5-K8 complex relative to apo C5**. Centre-of-mass translational (Å) and rotational (°) displacement when each C5-K8 domain is superimposed onto each apo C5 domain. The data for the C345C domain is absent as it could not be resolved in the crystal structure. The Cα root-mean-square deviation (RMSD) for each domain superimposition is shown below.

| **Complex** | **Metric** | **Apo C5** | | | | | | | | | | | |
| --- | --- | --- | --- | --- | --- | --- | --- | --- | --- | --- | --- | --- | --- |
|  |  | **MG1** | **MG2** | **MG3** | **MG4** | **MG5** | **MG6** | **Linker** | **C5a** | **MG7** | **CUB** | **C5d** | **MG8** |
| **MG1** | Distance (Å) | - | 1.5 | 12.6 | 3.0 | 1.0 | 2.6 | 0.6 | 4.9 | 3.5 | 4.6 | 2.4 | 3.8 |
| **MG1** | Angle (°) | - | 2.2 | 11.7 | 4.0 | 2.0 | 3.3 | 1.7 | 3.6 | 2.5 | 3.1 | 2.0 | 2.7 |
| **MG2** | Distance (Å) | 0.8 | - | 8.5 | 3.1 | 1.1 | 0.1 | 1.1 | 3.7 | 2.7 | 2.9 | 1.9 | 2.4 |
| **MG2** | Angle (°) | 0.9 | - | 13.1 | 3.5 | 1.3 | 0.2 | 1.6 | 3.9 | 3.4 | 3.3 | 3.0 | 2.7 |
| **MG3** | Distance (Å) | 5.1 | 4.4 | - | 0.9 | 3.9 | 3.4 | 5.2 | 3.8 | 3.9 | 5.6 | 5.4 | 5.6 |
| **MG3** | Angle (°) | 4.7 | 6.1 | - | 1.1 | 4.0 | 5.3 | 6.2 | 4.4 | 5.4 | 3.4 | 0.8 | 5.5 |
| **MG4** | Distance (Å) | 2.6 | 3.7 | 3.0 | - | 1.5 | 2.9 | 2.4 | 2.4 | 5.3 | 5.4 | 4.2 | 4.7 |
| **MG4** | Angle (°) | 3.5 | 4.3 | 4.3 | - | 3.5 | 3.6 | 5.6 | 2.0 | 4.3 | 3.2 | 2.2 | 3.4 |
| **MG5** | Distance (Å) | 1.0 | 2.3 | 8.3 | 1.2 | - | 2.0 | 0.8 | 4.2 | 5.4 | 4.3 | 2.0 | 3.0 |
| **MG5** | Angle (°) | 2.0 | 3.0 | 8.6 | 2.7 | - | 2.6 | 3.0 | 3.2 | 4.0 | 2.7 | 1.4 | 2.0 |
| **MG6** | Distance (Å) | 1.4 | 0.3 | 7.4 | 1.3 | 1.1 | - | 1.3 | 3.5 | 3.1 | 2.6 | 1.3 | 1.7 |
| **MG6** | Angle (°) | 1.7 | 0.8 | 11.6 | 1.6 | 1.3 | - | 1.7 | 3.2 | 5.0 | 2.6 | 1.1 | 1.1 |
| **Linker** | Distance (Å) | 0.5 | 1.7 | 8.2 | 1.0 | 0.4 | 1.9 | - | 3.3 | 3.6 | 4.4 | 2.2 | 3.4 |
| **Linker** | Angle (°) | 1.3 | 2.6 | 9.7 | 2.3 | 1.4 | 2.7 | - | 2.9 | 2.8 | 2.9 | 1.6 | 2.7 |
| **C5a** | Distance (Å) | 1.9 | 2.6 | 11.6 | 9.0 | 4.1 | 3.3 | 3.3 | - | 2.5 | 0.5 | 1.6 | 1.0 |
| **C5a** | Angle (°) | 0.4 | 1.4 | 22.9 | 8.5 | 3.1 | 2.9 | 2.8 | - | 2.9 | 0.2 | 2.0 | 1.6 |
| **MG7** | Distance (Å) | 4.0 | 1.2 | 5.3 | 3.0 | 4.0 | 1.7 | 4.8 | 1.0 | - | 1.6 | 1.1 | 0.8 |
| **MG7** | Angle (°) | 2.9 | 1.5 | 7.4 | 2.4 | 2.9 | 2.5 | 3.7 | 1.2 | - | 2.0 | 1.0 | 1.2 |
| **CUB** | Distance (Å) | 4.8 | 2.3 | 14.2 | 9.3 | 6.8 | 4.6 | 6.5 | 2.2 | 2.5 | - | 0.5 | 0.3 |
| **CUB** | Angle (°) | 3.3 | 2.6 | 11.9 | 5.7 | 4.2 | 4.8 | 4.4 | 2.0 | 3.3 | - | 0.9 | 0.5 |
| **C5d** | Distance (Å) | 3.8 | 2.8 | 16.5 | 10.2 | 6.1 | 4.5 | 5.5 | 3.3 | 2.8 | 0.1 | - | 0.1 |
| **C5d** | Angle (°) | 3.2 | 4.4 | 16.2 | 7.2 | 4.5 | 5.0 | 4.6 | 4.5 | 3.0 | 0.2 | - | 0.2 |
| **MG8** | Distance (Å) | 1.8 | 1.9 | 8.4 | 8.6 | 5.1 | 3.8 | 4.6 | 1.1 | 0.7 | 0.7 | 0.6 | - |
| **MG8** | Angle (°) | 1.3 | 1.7 | 10.7 | 6.4 | 3.5 | 4.1 | 3.6 | 1.9 | 1.1 | 1.2 | 1.1 | - |
| **Domain superimposition RMSD (Å)** | | 1.8 | 0.5 | 1.6 | 0.7 | 1.3 | 0.6 | 1.3 | 2.3 | 0.7 | 0.9 | 0.9 | 0.6 |

**Supplementary Table 10. Domain displacement in C5-K92 complex relative to apo C5**. Centre-of-mass translational (Å) and rotational (°) displacement when each C5-K92 domain is superimposed onto each apo C5 domain. The Cα root-mean-square deviation (RMSD) for each domain superimposition is shown below.

| **Complex** | **Metric** | **Apo C5** | | | | | | | | | | | | |
| --- | --- | --- | --- | --- | --- | --- | --- | --- | --- | --- | --- | --- | --- | --- |
|  |  | **MG1** | **MG2** | **MG3** | **MG4** | **MG5** | **MG6** | **Linker** | **C5a** | **MG7** | **CUB** | **C5d** | **MG8** | **C345C** |
| **MG1** | Distance (Å) | - | 0.7 | 11.3 | 2.3 | 1.0 | 1.5 | 2.6 | 13.9 | 7.0 | 7.5 | 4.9 | 2.1 | 117.3 |
| **MG1** | Angle (°) | - | 0.6 | 10.4 | 3.1 | 2.3 | 1.8 | 7.7 | 10.8 | 5.0 | 5.1 | 4.2 | 1.2 | 67.2 |
| **MG2** | Distance (Å) | 1.4 | - | 8.3 | 3.6 | 1.7 | 0.3 | 3.0 | 8.1 | 3.3 | 4.9 | 2.3 | 0.5 | 78.6 |
| **MG2** | Angle (°) | 1.9 | - | 12.9 | 4.3 | 2.0 | 0.9 | 5.0 | 10.6 | 4.2 | 5.5 | 3.6 | 0.2 | 68.1 |
| **MG3** | Distance (Å) | 3.7 | 3.9 | - | 1.4 | 2.5 | 3.2 | 8.6 | 5.1 | 5.8 | 5.0 | 5.3 | 4.6 | 103.7 |
| **MG3** | Angle (°) | 3.3 | 5.4 | - | 2.1 | 2.6 | 5.2 | 10.5 | 9.4 | 8.0 | 3.6 | 4.2 | 3.8 | 117.1 |
| **MG4** | Distance (Å) | 1.6 | 3.5 | 2.0 | - | 1.2 | 2.4 | 4.7 | 14.3 | 9.5 | 7.6 | 8.5 | 6.2 | 151.7 |
| **MG4** | Angle (°) | 2.2 | 4.1 | 2.9 | - | 2.6 | 3.0 | 11.1 | 13.5 | 7.6 | 4.7 | 6.2 | 4.2 | 104.0 |
| **MG5** | Distance (Å) | 0.8 | 2.1 | 7.3 | 1.1 | - | 0.9 | 2.3 | 16.8 | 9.2 | 8.2 | 7.8 | 4.6 | 132.7 |
| **MG5** | Angle (°) | 1.9 | 2.7 | 7.5 | 2.6 | - | 1.2 | 8.3 | 13.0 | 6.7 | 5.0 | 5.7 | 2.9 | 78.2 |
| **MG6** | Distance (Å) | 2.4 | 0.2 | 7.1 | 2.5 | 1.0 | - | 2.0 | 10.6 | 3.0 | 4.9 | 2.8 | 1.1 | 61.0 |
| **MG6** | Angle (°) | 2.9 | 0.4 | 11.5 | 3.1 | 1.3 | - | 2.8 | 11.7 | 4.7 | 5.0 | 3.2 | 1.3 | 55.2 |
| **Linker** | Distance (Å) | 1.0 | 1.4 | 6.6 | 1.0 | 0.5 | 0.8 | - | 12.7 | 7.3 | 7.6 | 5.7 | 3.1 | 129.5 |
| **Linker** | Angle (°) | 2.6 | 1.7 | 8.0 | 1.9 | 1.3 | 1.0 | - | 11.8 | 5.8 | 5.3 | 4.9 | 1.9 | 82.9 |
| **C5a** | Distance (Å) | 2.7 | 0.7 | 8.7 | 6.3 | 4.3 | 2.0 | 7.8 | - | 3.8 | 3.8 | 2.9 | 1.9 | 107.6 |
| **C5a** | Angle (°) | 2.0 | 0.9 | 18.2 | 5.5 | 3.2 | 2.2 | 7.2 | - | 4.6 | 3.5 | 3.8 | 2.9 | 133.3 |
| **MG7** | Distance (Å) | 3.0 | 0.6 | 4.5 | 3.9 | 3.6 | 1.8 | 6.4 | 9.5 | - | 3.7 | 1.0 | 1.3 | 27.9 |
| **MG7** | Angle (°) | 2.1 | 0.7 | 6.1 | 3.1 | 2.7 | 2.7 | 5.1 | 11.3 | - | 5.0 | 0.8 | 2.1 | 42.0 |
| **CUB** | Distance (Å) | 1.1 | 2.1 | 14.7 | 11.3 | 7.1 | 4.0 | 7.1 | 7.6 | 0.8 | - | 0.8 | 2.1 | 59.2 |
| **CUB** | Angle (°) | 0.7 | 2.2 | 12.7 | 7.0 | 4.4 | 4.1 | 5.0 | 7.3 | 1.0 | - | 1.2 | 3.4 | 72.5 |
| **C5d** | Distance (Å) | 0.9 | 1.0 | 14.9 | 9.4 | 5.1 | 2.2 | 7.8 | 3.9 | 1.8 | 2.9 | - | 1.2 | 92.8 |
| **C5d** | Angle (°) | 0.3 | 1.6 | 15.4 | 6.8 | 3.7 | 2.5 | 6.8 | 5.2 | 1.8 | 5.6 | - | 2.1 | 94.0 |
| **MG8** | Distance (Å) | 1.7 | 1.4 | 8.0 | 7.9 | 5.9 | 3.3 | 8.8 | 5.2 | 1.1 | 1.4 | 0.5 | - | 63.4 |
| **MG8** | Angle (°) | 0.8 | 1.6 | 10.1 | 5.6 | 3.9 | 3.8 | 6.7 | 10.6 | 1.7 | 2.2 | 0.8 | - | 105.8 |
| **C345C** | Distance (Å) | 39.9 | 40.6 | 40.8 | 43.0 | 43.9 | 42.5 | 35.1 | 46.2 | 40.0 | 38.0 | 40.7 | 42.1 | - |
| **C345C** | Angle (°) | 20.6 | 32.7 | 28.7 | 19.8 | 21.3 | 36.3 | 17.4 | 43.5 | 70.9 | 40.3 | 36.9 | 63.8 | - |
| **Domain superimposition RMSD (Å)** | | 1.7 | 0.5 | 1.4 | 0.8 | 1.4 | 0.7 | 2.8 | 4.8 | 1.1 | 1.0 | 1.1 | 1.7 | 2.5 |

**Supplementary Table 11. Domain displacement in C5-SSL7 complex relative to apo C5**. Centre-of-mass translational (Å) and rotational (°) displacement when each C5-SSL7 domain is superimposed onto each apo C5 domain. The Cα root-mean-square deviation (RMSD) for each domain superimposition is shown below.

| **Complex** | **Metric** | **Apo C5** | | | | | | | | | | | | |
| --- | --- | --- | --- | --- | --- | --- | --- | --- | --- | --- | --- | --- | --- | --- |
|  |  | **MG1** | **MG2** | **MG3** | **MG4** | **MG5** | **MG6** | **Linker** | **C5a** | **MG7** | **CUB** | **C5d** | **MG8** | **C345C** |
| **MG1** | Distance (Å) | - | 0.6 | 0.9 | 0.5 | 0.4 | 0.1 | 0.6 | 1.2 | 1.4 | 0.5 | 0.5 | 1.1 | 1.6 |
| **MG1** | Angle (°) | - | 0.9 | 0.8 | 0.4 | 0.9 | 0.0 | 1.5 | 0.9 | 1.0 | 0.3 | 0.4 | 0.8 | 0.9 |
| **MG2** | Distance (Å) | 0.9 | - | 0.2 | 0.1 | 0.5 | 0.2 | 0.3 | 0.7 | 0.5 | 0.3 | 0.2 | 0.5 | 0.8 |
| **MG2** | Angle (°) | 1.3 | - | 0.3 | 0.1 | 0.6 | 0.5 | 0.5 | 0.6 | 0.7 | 0.3 | 0.3 | 0.6 | 0.7 |
| **MG3** | Distance (Å) | 1.0 | 0.6 | - | 0.2 | 1.3 | 0.3 | 0.8 | 0.3 | 0.7 | 1.1 | 0.7 | 0.6 | 0.6 |
| **MG3** | Angle (°) | 1.0 | 1.1 | - | 0.3 | 1.3 | 0.3 | 1.0 | 0.8 | 0.9 | 1.0 | 0.7 | 1.0 | 0.7 |
| **MG4** | Distance (Å) | 0.5 | 1.1 | 0.3 | - | 0.4 | 0.3 | 0.2 | 0.7 | 1.0 | 1.1 | 0.4 | 0.8 | 1.0 |
| **MG4** | Angle (°) | 0.4 | 1.3 | 0.5 | - | 0.7 | 0.4 | 0.4 | 0.6 | 0.8 | 0.7 | 0.3 | 0.6 | 0.7 |
| **MG5** | Distance (Å) | 0.3 | 1.0 | 0.9 | 0.3 | - | 0.3 | 0.2 | 1.0 | 1.2 | 0.8 | 0.2 | 1.0 | 1.3 |
| **MG5** | Angle (°) | 0.7 | 1.3 | 0.9 | 0.4 | - | 0.2 | 0.5 | 0.6 | 0.8 | 0.5 | 0.1 | 0.6 | 0.7 |
| **MG6** | Distance (Å) | 1.0 | 0.3 | 0.4 | 0.2 | 0.8 | - | 0.5 | 0.7 | 0.4 | 0.5 | 0.4 | 0.3 | 0.6 |
| **MG6** | Angle (°) | 1.2 | 0.9 | 0.5 | 0.1 | 0.9 | - | 0.8 | 0.5 | 0.6 | 0.5 | 0.4 | 0.4 | 0.6 |
| **Linker** | Distance (Å) | 0.3 | 0.7 | 0.6 | 0.1 | 0.2 | 0.3 | - | 0.8 | 0.9 | 0.7 | 0.1 | 0.8 | 1.1 |
| **Linker** | Angle (°) | 0.5 | 1.4 | 0.7 | 0.2 | 0.6 | 0.3 | - | 0.7 | 0.8 | 0.5 | 0.1 | 0.6 | 0.7 |
| **C5a** | Distance (Å) | 1.5 | 0.8 | 0.9 | 0.7 | 1.3 | 0.6 | 0.9 | - | 0.4 | 0.9 | 0.6 | 0.3 | 0.4 |
| **C5a** | Angle (°) | 1.1 | 0.8 | 2.1 | 0.6 | 1.0 | 0.4 | 0.8 | - | 0.3 | 0.9 | 0.8 | 0.5 | 0.4 |
| **MG7** | Distance (Å) | 1.8 | 0.5 | 0.8 | 0.2 | 1.3 | 0.3 | 0.8 | 0.4 | - | 0.4 | 0.3 | 0.1 | 0.4 |
| **MG7** | Angle (°) | 1.3 | 0.6 | 1.0 | 0.1 | 0.9 | 0.5 | 0.6 | 0.3 | - | 0.5 | 0.3 | 0.2 | 0.9 |
| **CUB** | Distance (Å) | 2.8 | 0.7 | 0.2 | 0.3 | 1.4 | 0.8 | 0.8 | 0.4 | 0.4 | - | 0.1 | 0.2 | 0.7 |
| **CUB** | Angle (°) | 1.9 | 0.8 | 0.2 | 0.2 | 0.8 | 0.8 | 0.6 | 0.3 | 0.5 | - | 0.1 | 0.4 | 0.7 |
| **C5d** | Distance (Å) | 2.3 | 0.3 | 0.5 | 0.6 | 1.2 | 0.7 | 0.7 | 0.3 | 0.4 | 0.2 | - | 0.2 | 0.6 |
| **C5d** | Angle (°) | 2.0 | 0.5 | 0.5 | 0.4 | 0.8 | 0.8 | 0.7 | 0.3 | 0.4 | 0.4 | - | 0.3 | 0.5 |
| **MG8** | Distance (Å) | 1.9 | 0.6 | 0.6 | 0.4 | 1.3 | 0.2 | 0.8 | 0.2 | 0.2 | 0.3 | 0.2 | - | 0.5 |
| **MG8** | Angle (°) | 1.3 | 0.8 | 0.9 | 0.3 | 0.9 | 0.2 | 0.6 | 0.3 | 0.3 | 0.5 | 0.4 | - | 0.6 |
| **C345C** | Distance (Å) | 2.2 | 0.2 | 0.8 | 0.2 | 1.9 | 0.6 | 1.2 | 0.6 | 0.4 | 0.8 | 0.7 | 0.4 | - |
| **C345C** | Angle (°) | 1.3 | 0.1 | 1.0 | 0.0 | 1.2 | 0.7 | 0.8 | 0.6 | 0.9 | 0.8 | 0.6 | 0.3 | - |
| **Domain superimposition RMSD (Å)** | | 1.0 | 0.3 | 0.3 | 0.3 | 0.3 | 0.3 | 0.8 | 0.4 | 0.5 | 0.3 | 0.5 | 0.3 | 1.4 |

**Supplementary Table 12. Domain displacement in C5-SSL7 β-grasp domain complex relative to apo C5**. Centre-of-mass translational (Å) and rotational (°) displacement when each C5-SSL7 β-grasp domain complex domain is superimposed onto each apo C5 domain. The data for the C345C domain is absent as it could not be resolved in the crystal structure. The Cα root-mean-square deviation (RMSD) for each domain superimposition is shown below.

| **Complex** | **Metric** | **Apo C5** | | | | | | | | | | | |
| --- | --- | --- | --- | --- | --- | --- | --- | --- | --- | --- | --- | --- | --- |
|  |  | **MG1** | **MG2** | **MG3** | **MG4** | **MG5** | **MG6** | **Linker** | **C5a** | **MG7** | **CUB** | **C5d** | **MG8** |
| **MG1** | Distance (Å) | - | 0.8 | 0.9 | 0.5 | 0.4 | 0.0 | 0.5 | 1.5 | 1.2 | 0.4 | 0.8 | 1.6 |
| **MG1** | Angle (°) | - | 1.2 | 0.9 | 0.7 | 0.9 | 0.0 | 1.4 | 1.0 | 0.9 | 0.2 | 0.7 | 1.1 |
| **MG2** | Distance (Å) | 0.6 | - | 0.4 | 0.4 | 0.6 | 0.2 | 0.3 | 1.0 | 0.5 | 0.1 | 0.4 | 0.9 |
| **MG2** | Angle (°) | 0.9 | - | 0.6 | 0.5 | 0.7 | 0.6 | 0.6 | 0.7 | 0.6 | 0.1 | 0.6 | 1.0 |
| **MG3** | Distance (Å) | 0.9 | 1.2 | - | 0.4 | 1.3 | 0.6 | 1.0 | 0.7 | 0.8 | 1.5 | 1.2 | 1.1 |
| **MG3** | Angle (°) | 0.9 | 2.0 | - | 0.5 | 1.3 | 0.8 | 1.3 | 1.4 | 1.1 | 1.3 | 1.0 | 1.6 |
| **MG4** | Distance (Å) | 0.2 | 1.8 | 0.2 | - | 0.5 | 0.3 | 0.3 | 0.8 | 1.0 | 1.6 | 1.0 | 1.3 |
| **MG4** | Angle (°) | 0.1 | 2.3 | 0.4 | - | 1.1 | 0.4 | 0.7 | 0.7 | 0.8 | 1.0 | 0.6 | 1.0 |
| **MG5** | Distance (Å) | 0.3 | 1.3 | 0.7 | 0.3 | - | 0.2 | 0.2 | 1.2 | 1.0 | 0.9 | 0.4 | 1.3 |
| **MG5** | Angle (°) | 0.7 | 1.8 | 0.7 | 0.3 | - | 0.1 | 0.8 | 0.8 | 0.7 | 0.6 | 0.3 | 0.9 |
| **MG6** | Distance (Å) | 0.6 | 0.5 | 0.5 | 0.3 | 0.8 | - | 0.5 | 0.9 | 0.4 | 0.6 | 0.2 | 0.6 |
| **MG6** | Angle (°) | 0.7 | 1.3 | 0.7 | 0.4 | 1.0 | - | 0.7 | 0.7 | 0.6 | 0.6 | 0.2 | 0.6 |
| **Linker** | Distance (Å) | 0.3 | 1.0 | 0.6 | 0.2 | 0.3 | 0.2 | - | 1.0 | 0.8 | 0.8 | 0.5 | 1.2 |
| **Linker** | Angle (°) | 0.7 | 2.0 | 0.7 | 0.3 | 0.8 | 0.3 | - | 0.9 | 0.7 | 0.6 | 0.4 | 1.0 |
| **C5a** | Distance (Å) | 1.6 | 1.0 | 1.4 | 1.6 | 1.7 | 0.8 | 1.3 | - | 0.4 | 1.1 | 0.7 | 0.4 |
| **C5a** | Angle (°) | 1.2 | 0.8 | 3.1 | 1.6 | 1.2 | 0.6 | 1.2 | - | 0.4 | 1.0 | 0.9 | 0.8 |
| **MG7** | Distance (Å) | 1.3 | 0.4 | 0.8 | 0.5 | 1.4 | 0.4 | 1.0 | 0.6 | - | 0.5 | 0.4 | 0.2 |
| **MG7** | Angle (°) | 0.9 | 0.5 | 1.0 | 0.4 | 1.0 | 0.6 | 0.8 | 0.6 | - | 0.7 | 0.4 | 0.3 |
| **CUB** | Distance (Å) | 2.5 | 0.6 | 1.0 | 1.1 | 1.9 | 1.0 | 1.5 | 0.6 | 0.5 | - | 0.1 | 0.4 |
| **CUB** | Angle (°) | 1.7 | 0.7 | 0.9 | 0.7 | 1.2 | 1.0 | 1.0 | 0.5 | 0.6 | - | 0.3 | 0.6 |
| **C5d** | Distance (Å) | 2.3 | 0.4 | 1.4 | 1.6 | 1.7 | 1.0 | 1.4 | 0.3 | 0.5 | 0.2 | - | 0.1 |
| **C5d** | Angle (°) | 2.0 | 0.7 | 1.3 | 1.1 | 1.3 | 1.2 | 1.3 | 0.5 | 0.5 | 0.4 | - | 0.3 |
| **MG8** | Distance (Å) | 1.6 | 0.6 | 0.8 | 1.4 | 1.6 | 0.4 | 1.2 | 0.2 | 0.4 | 0.4 | 0.2 | - |
| **MG8** | Angle (°) | 1.2 | 0.7 | 1.0 | 1.1 | 1.1 | 0.5 | 1.0 | 0.3 | 0.7 | 0.6 | 0.3 | - |
| **Domain superimposition RMSD (Å)** | | 1.0 | 0.4 | 0.5 | 0.4 | 0.4 | 0.4 | 0.8 | 0.5 | 0.6 | 0.3 | 0.5 | 0.4 |

**Supplementary Table 13. Rigidity retention in C5 structures at -0.5 kcal/mol energy cut-off**. The size and persistence of rigid clusters in each C5 domain at -0.5 kcal/mol across C5 structures, shown as the percentage of residues within the domain whose Cα is part of one of the twenty largest rigid clusters. The data for the C345C domain is absent for C5-CVF-H1H, C5-K8, and C5-SSL7 β-grasp domain complexes as it could not be resolved in the crystal structure. Values are colour-coded from light blue (1-49%) to purple (≥50%), with intensity proportional to the magnitude of retention. Rigid cluster decomposition (RCD) was conducted using the FLEXOME software suite (18).

| **Domain** | **Apo C5** | **C5-CVF** | **C5-CVF-SSL7** | **C5-CVF-H1H** | **C5-OmCI-RaCI1** | **C5-OmCI-RaCI2** | **C5-OmCI-RaCI3** | **C5-OmCI-RaCI1-CirpT1** | **C5-Eculizumab** | **C5-K8** | **C5-K92** | **C5-SSL7** | **C5-SSL7 β-grasp domain** |
| --- | --- | --- | --- | --- | --- | --- | --- | --- | --- | --- | --- | --- | --- |
| **MG1** | 0% | 26% | 0% | 0% | 85% | 85% | 85% | 0% | 0% | 82% | 88% | 32% | 1% |
| **MG2** | 79% | 6% | 0% | 91% | 96% | 96% | 96% | 84% | 75% | 96% | 96% | 11% | 10% |
| **MG3** | 9% | 6% | 0% | 18% | 65% | 76% | 61% | 0% | 2% | 10% | 16% | 8% | 8% |
| **MG4** | 75% | 69% | 43% | 5% | 45% | 44% | 44% | 0% | 84% | 94% | 71% | 12% | 5% |
| **MG5** | 25% | 0% | 25% | 25% | 68% | 67% | 32% | 0% | 26% | 83% | 36% | 0% | 0% |
| **MG6** | 0% | 5% | 6% | 25% | 87% | 39% | 90% | 0% | 49% | 62% | 86% | 0% | 0% |
| **Linker** | 0% | 17% | 37% | 19% | 50% | 39% | 37% | 20% | 39% | 88% | 62% | 38% | 35% |
| **C5a** | 36% | 54% | 65% | 41% | 69% | 74% | 73% | 59% | 64% | 81% | 71% | 63% | 51% |
| **MG7** | 24% | 0% | 32% | 0% | 4% | 4% | 83% | 0% | 0% | 4% | 89% | 0% | 0% |
| **CUB** | 0% | 0% | 0% | 0% | 5% | 6% | 10% | 0% | 0% | 40% | 76% | 11% | 0% |
| **C5d** | 64% | 72% | 63% | 49% | 89% | 89% | 89% | 56% | 62% | 92% | 89% | 70% | 64% |
| **MG8** | 7% | 75% | 7% | 61% | 74% | 77% | 83% | 6% | 66% | 84% | 84% | 9% | 63% |
| **C345C** | 11% | 12% | 13% | - | 59% | 61% | 60% | 53% | 45% | - | 59% | 13% | - |
| **Total** | **30%** | **32%** | **26%** | **31%** | **65%** | **63%** | **69%** | **25%** | **42%** | **70%** | **73%** | **25%** | **26%** |
| **Total without C345C** | **32%** | **34%** | **27%** | **31%** | **66%** | **63%** | **69%** | **22%** | **42%** | **70%** | **74%** | **27%** | **26%** |

**Supplementary Table 14. Rigidity retention in C5 structures at -1.0 kcal/mol energy cut-off**. The size and persistence of rigid clusters in each C5 domain at -1.0 kcal/mol across C5 structures, shown as the percentage of residues within the domain whose Cα is part of one of the twenty largest rigid clusters. The data for the C345C domain is absent for C5-CVF-H1H, C5-K8, and C5-SSL7 β-grasp domain complexes as it could not be resolved in the crystal structure. Values are colour-coded from light blue (1-49%) to purple (≥50%), with intensity proportional to the magnitude of retention. Rigid cluster decomposition (RCD) was conducted using the FLEXOME software suite (18).

| **Domain** | **Apo C5** | **C5-CVF** | **C5-CVF-SSL7** | **C5-CVF-H1H** | **C5-OmCI-RaCI1** | **C5-OmCI-RaCI2** | **C5-OmCI-RaCI3** | **C5-OmCI-RaCI1-CirpT1** | **C5-Eculizumab** | **C5-K8** | **C5-K92** | **C5-SSL7** | **C5-SSL7 β-grasp domain** |
| --- | --- | --- | --- | --- | --- | --- | --- | --- | --- | --- | --- | --- | --- |
| **MG1** | 6% | 6% | 0% | 0% | 40% | 40% | 43% | 0% | 0% | 82% | 88% | 8% | 1% |
| **MG2** | 6% | 0% | 6% | 0% | 12% | 12% | 96% | 5% | 0% | 96% | 88% | 11% | 10% |
| **MG3** | 0% | 5% | 0% | 0% | 5% | 9% | 57% | 0% | 0% | 9% | 3% | 5% | 8% |
| **MG4** | 5% | 0% | 0% | 0% | 44% | 44% | 44% | 0% | 0% | 92% | 6% | 8% | 0% |
| **MG5** | 0% | 0% | 0% | 0% | 31% | 28% | 28% | 0% | 26% | 36% | 28% | 4% | 4% |
| **MG6** | 0% | 5% | 0% | 4% | 4% | 0% | 4% | 0% | 0% | 9% | 6% | 2% | 0% |
| **Linker** | 13% | 15% | 12% | 15% | 19% | 19% | 19% | 18% | 21% | 65% | 51% | 28% | 17% |
| **C5a** | 34% | 41% | 50% | 32% | 68% | 68% | 68% | 48% | 54% | 64% | 67% | 45% | 31% |
| **MG7** | 0% | 0% | 15% | 0% | 0% | 0% | 4% | 0% | 0% | 4% | 4% | 0% | 0% |
| **CUB** | 3% | 0% | 0% | 0% | 0% | 0% | 0% | 0% | 0% | 3% | 3% | 0% | 0% |
| **C5d** | 58% | 58% | 60% | 42% | 85% | 87% | 86% | 54% | 59% | 90% | 70% | 66% | 59% |
| **MG8** | 7% | 7% | 7% | 2% | 69% | 76% | 69% | 6% | 0% | 80% | 70% | 8% | 8% |
| **C345C** | 6% | 7% | 11% | - | 48% | 55% | 54% | 45% | 17% | - | 57% | 0% | - |
| **Total** | **16%** | **16%** | **18%** | **12%** | **40%** | **42%** | **51%** | **19%** | **19%** | **57%** | **45%** | **20%** | **18%** |
| **Total without C345C** | **17%** | **17%** | **18%** | **12%** | **40%** | **40%** | **50%** | **16%** | **19%** | **57%** | **43%** | **22%** | **18%** |

**Supplementary Table 15. Rigidity retention in C5 structures at -1.5 kcal/mol energy cut-off**. The size and persistence of rigid clusters in each C5 domain at -1.5 kcal/mol across C5 structures, shown as the percentage of residues within the domain whose Cα is part of one of the twenty largest rigid clusters. The data for the C345C domain is absent for C5-CVF-H1H, C5-K8, and C5-SSL7 β-grasp domain complexes as it could not be resolved in the crystal structure. Values are colour-coded from light blue (1-49%) to purple (≥50%), with intensity proportional to the magnitude of retention. Rigid cluster decomposition (RCD) was conducted using the FLEXOME software suite (18).

| **Domain** | **Apo C5** | **C5-CVF** | **C5-CVF-SSL7** | **C5-CVF-H1H** | **C5-OmCI-RaCI1** | **C5-OmCI-RaCI2** | **C5-OmCI-RaCI3** | **C5-OmCI-RaCI1-CirpT1** | **C5-Eculizumab** | **C5-K8** | **C5-K92** | **C5-SSL7** | **C5-SSL7 β-grasp domain** |
| --- | --- | --- | --- | --- | --- | --- | --- | --- | --- | --- | --- | --- | --- |
| **MG1** | 0% | 6% | 0% | 0% | 22% | 0% | 37% | 0% | 0% | 59% | 0% | 0% | 1% |
| **MG2** | 0% | 0% | 6% | 0% | 12% | 7% | 89% | 0% | 0% | 5% | 72% | 11% | 3% |
| **MG3** | 0% | 0% | 0% | 0% | 0% | 0% | 0% | 0% | 0% | 3% | 0% | 5% | 0% |
| **MG4** | 2% | 4% | 0% | 2% | 0% | 40% | 2% | 2% | 0% | 6% | 0% | 5% | 8% |
| **MG5** | 0% | 0% | 0% | 2% | 31% | 28% | 28% | 0% | 26% | 0% | 0% | 4% | 4% |
| **MG6** | 0% | 0% | 0% | 2% | 4% | 0% | 6% | 0% | 0% | 9% | 0% | 2% | 0% |
| **Linker** | 13% | 15% | 0% | 9% | 13% | 13% | 13% | 14% | 12% | 44% | 19% | 15% | 17% |
| **C5a** | 25% | 36% | 45% | 12% | 54% | 46% | 63% | 44% | 53% | 59% | 38% | 16% | 17% |
| **MG7** | 0% | 0% | 15% | 0% | 0% | 0% | 4% | 0% | 0% | 4% | 0% | 0% | 0% |
| **CUB** | 3% | 0% | 0% | 0% | 0% | 0% | 0% | 0% | 0% | 0% | 0% | 0% | 0% |
| **C5d** | 54% | 55% | 60% | 32% | 69% | 60% | 77% | 50% | 51% | 85% | 60% | 53% | 49% |
| **MG8** | 4% | 6% | 7% | 2% | 63% | 69% | 65% | 6% | 0% | 41% | 65% | 6% | 8% |
| **C345C** | 6% | 7% | 9% | - | 28% | 45% | 41% | 9% | 9% | - | 56% | 0% | - |
| **Total** | **14%** | **15%** | **17%** | **9%** | **29%** | **30%** | **39%** | **14%** | **16%** | **33%** | **30%** | **14%** | **14%** |
| **Total without C345C** | **15%** | **16%** | **18%** | **9%** | **30%** | **28%** | **39%** | **15%** | **16%** | **33%** | **27%** | **16%** | **14%** |

**Supplementary Table 16. Rigidity retention in C5 structures at -2.0 kcal/mol energy cut-off**. The size and persistence of rigid clusters in each C5 domain at -2.0 kcal/mol across C5 structures, shown as the percentage of residues within the domain whose Cα is part of one of the twenty largest rigid clusters. The data for the C345C domain is absent for C5-CVF-H1H, C5-K8, and C5-SSL7 β-grasp domain complexes as it could not be resolved in the crystal structure. Values are colour-coded from light blue (1-49%) to purple (≥50%), with intensity proportional to the magnitude of retention. Rigid cluster decomposition (RCD) was conducted using the FLEXOME software suite (18).

| **Domain** | **Apo C5** | **C5-CVF** | **C5-CVF-SSL7** | **C5-CVF-H1H** | **C5-OmCI-RaCI1** | **C5-OmCI-RaCI2** | **C5-OmCI-RaCI3** | **C5-OmCI-RaCI1-CirpT1** | **C5-Eculizumab** | **C5-K8** | **C5-K92** | **C5-SSL7** | **C5-SSL7 β-grasp domain** |
| --- | --- | --- | --- | --- | --- | --- | --- | --- | --- | --- | --- | --- | --- |
| **MG1** | 0% | 0% | 0% | 0% | 0% | 0% | 34% | 0% | 0% | 12% | 0% | 0% | 0% |
| **MG2** | 0% | 3% | 2% | 3% | 0% | 5% | 70% | 0% | 5% | 5% | 0% | 7% | 9% |
| **MG3** | 0% | 0% | 4% | 0% | 0% | 0% | 0% | 0% | 0% | 0% | 0% | 0% | 0% |
| **MG4** | 2% | 4% | 0% | 0% | 0% | 0% | 0% | 2% | 2% | 4% | 0% | 0% | 4% |
| **MG5** | 0% | 0% | 4% | 2% | 31% | 28% | 28% | 0% | 0% | 0% | 0% | 0% | 4% |
| **MG6** | 2% | 0% | 2% | 2% | 3% | 0% | 0% | 0% | 0% | 0% | 0% | 2% | 2% |
| **Linker** | 12% | 15% | 0% | 0% | 13% | 13% | 13% | 0% | 12% | 26% | 19% | 15% | 15% |
| **C5a** | 25% | 34% | 31% | 0% | 46% | 43% | 36% | 44% | 22% | 59% | 30% | 18% | 17% |
| **MG7** | 0% | 4% | 4% | 0% | 0% | 0% | 0% | 0% | 0% | 0% | 4% | 0% | 0% |
| **CUB** | 3% | 0% | 0% | 0% | 0% | 0% | 0% | 0% | 2% | 0% | 0% | 0% | 0% |
| **C5d** | 48% | 52% | 52% | 15% | 55% | 54% | 53% | 48% | 49% | 61% | 53% | 49% | 36% |
| **MG8** | 4% | 0% | 0% | 2% | 49% | 0% | 0% | 7% | 0% | 30% | 40% | 7% | 0% |
| **C345C** | 0% | 7% | 8% | - | 8% | 45% | 37% | 7% | 9% | - | 53% | 0% | - |
| **Total** | **12%** | **14%** | **14%** | **4%** | **21%** | **20%** | **25%** | **13%** | **13%** | **21%** | **21%** | **12%** | **11%** |
| **Total without C345C** | **13%** | **14%** | **14%** | **4%** | **22%** | **17%** | **23%** | **14%** | **13%** | **21%** | **18%** | **14%** | **11%** |
